# A systematic review and meta-analysis of randomized trials of substituting soymilk for cow’s milk and intermediate cardiometabolic outcomes: understanding the impact of dairy alternatives in the transition to plant-based diets on cardiometabolic health

**DOI:** 10.1186/s12916-024-03524-7

**Published:** 2024-08-22

**Authors:** M. N. Erlich, D. Ghidanac, S. Blanco Mejia, T. A. Khan, L. Chiavaroli, A. Zurbau, S. Ayoub-Charette, A. Almneni, M. Messina, L. A. Leiter, R. P. Bazinet, D. J. A. Jenkins, C. W. C. Kendall, J. L. Sievenpiper

**Affiliations:** 1 Department of Nutritional Sciences, Temerty Faculty of Medicine, University of Toronto, Toronto, ON Canada; 2 Toronto 3D Knowledge Synthesis and Clinical Trials Unit, Clinical Nutrition and Risk Factor Modification Centre, St. Michael’s Hospital, Toronto, ON Canada; 3 Li Ka Shing Knowledge Institute, St. Michael’s Hospital, Toronto, ON Canada; 4 Royal College of Surgeons in Ireland, Dublin, Ireland; 5 Soy Nutrition Institute Global, Washington, DC USA; 6 Department of Medicine, Temerty Faculty of Medicine, University of Toronto, Toronto, ON Canada; 7 Division of Endocrinology and Metabolism, Department of Medicine, St. Michael’s Hospital, Toronto, ON Canada; 8 College of Pharmacy and Nutrition, University of Saskatchewan, Saskatoon, SK Canada

**Keywords:** Soy protein, Soymilk, Cardiovascular disease, Systematic review, Meta-analysis, Randomized controlled feeding trials

## Abstract

**Background:**

Dietary guidelines recommend a shift to plant-based diets. Fortified soymilk, a prototypical plant protein food used in the transition to plant-based diets, usually contains added sugars to match the sweetness of cow’s milk and is classified as an ultra-processed food. Whether soymilk can replace minimally processed cow’s milk without the adverse cardiometabolic effects attributed to added sugars and ultra-processed foods remains unclear. We conducted a systematic review and meta-analysis of randomized controlled trials, to assess the effect of substituting soymilk for cow’s milk and its modification by added sugars (sweetened versus unsweetened) on intermediate cardiometabolic outcomes.

**Methods:**

MEDLINE, Embase, and the Cochrane Central Register of Controlled Trials were searched (through June 2024) for randomized controlled trials of ≥ 3 weeks in adults. Outcomes included established markers of blood lipids, glycemic control, blood pressure, inflammation, adiposity, renal disease, uric acid, and non-alcoholic fatty liver disease. Two independent reviewers extracted data and assessed risk of bias. The certainty of evidence was assessed using GRADE (Grading of Recommendations, Assessment, Development, and Evaluation). A sub-study of lactose versus sucrose outside of a dairy-like matrix was conducted to explore the role of sweetened soymilk which followed the same methodology.

**Results:**

Eligibility criteria were met by 17 trials (*n* = 504 adults with a range of health statuses), assessing the effect of a median daily dose of 500 mL of soymilk (22 g soy protein and 17.2 g or 6.9 g/250 mL added sugars) in substitution for 500 mL of cow’s milk (24 g milk protein and 24 g or 12 g/250 mL total sugars as lactose) on 19 intermediate outcomes. The substitution of soymilk for cow’s milk resulted in moderate reductions in non-HDL-C (mean difference, − 0.26 mmol/L [95% confidence interval, − 0.43 to − 0.10]), systolic blood pressure (− 8.00 mmHg [− 14.89 to − 1.11]), and diastolic blood pressure (− 4.74 mmHg [− 9.17 to − 0.31]); small important reductions in LDL-C (− 0.19 mmol/L [− 0.29 to − 0.09]) and c-reactive protein (CRP) (− 0.82 mg/L [− 1.26 to − 0.37]); and trivial increases in HDL-C (0.05 mmol/L [0.00 to 0.09]). No other outcomes showed differences. There was no meaningful effect modification by added sugars across outcomes. The certainty of evidence was high for LDL-C and non-HDL-C; moderate for systolic blood pressure, diastolic blood pressure, CRP, and HDL-C; and generally moderate-to-low for all other outcomes. We could not conduct the sub-study of the effect of lactose versus added sugars, as no eligible trials could be identified.

**Conclusions:**

Current evidence provides a good indication that replacing cow’s milk with soymilk (including sweetened soymilk) does not adversely affect established cardiometabolic risk factors and may result in advantages for blood lipids, blood pressure, and inflammation in adults with a mix of health statuses. The classification of plant-based dairy alternatives such as soymilk as ultra-processed may be misleading as it relates to their cardiometabolic effects and may need to be reconsidered in the transition to plant-based diets.

**Trial registration:**

ClinicalTrials.gov identifier, NCT05637866.

**Supplementary Information:**

The online version contains supplementary material available at 10.1186/s12916-024-03524-7.

## Background

Major dietary guidelines recommend a shift to plant-based diets for public and planetary health [1–8], while recommending simultaneous reductions in ultra-processed foods [2–8]. The shift to plant-based diets has resulted in an explosion of dairy, meat, and egg alternatives with plant protein foods projected to reach almost 10% of the global protein market by 2030 [9]. Although these foods can aid in the transition to plant-based diets, food classification systems such as the World Health Organization (WHO)-endorsed NOVA classification system classify them as ultra-processed foods to be avoided [10].

Dairy alternatives are an important example of a food category at the crossroads of these competing recommendations. School milk programs provide > 150 million servings of cow’s milk to children worldwide [11]. These programs are in addition to the food service and procurement policies of public institutions such as schools, universities, hospitals, long-term care homes, and prisons. Many of these programs and policies do not allow for the free replacement of cow’s milk with nutrient-dense plant milks [12, 13]. Although the Dietary Guidelines for Americans [1], Canada’s Food Guide [3], and several European food-based dietary guidelines [14] recognize fortified soymilk [1] as nutritionally equivalent to cow’s milk, school nutrition programs in the United States (US) [12] and Europe [13] only provide funding for cow’s milk. There is a bipartisan bill before the US congress to change this policy and provide funding for fortified soymilk [15]. A major barrier to the use of fortified soymilk is that it contains added sugars to match the sweetness of cow’s milk at a level which would disqualify it from meeting the Food and Drug Administration’s proposed definition of “healthy” [16] (although its total sugar content is usually ~ 60% less than that of cow’s milk given the higher sweetness intensity of sucrose vs lactose) [17] and is classified (irrespective of its sugar content) as an ultra-processed food to be avoided [10, 18]. Cow’s milk, on the other hand, enjoys classification as a “healthy,” minimally processed food to be encouraged [10, 18].

As industry innovates in response to the growing demand and policy makers develop public health nutrition policies and programs in response to the evolving dietary guidance for more plant-based diets, it is important to understand whether nutrient-dense ultra-processed plant protein foods can replace minimally processed dairy foods without the adverse cardiometabolic effects attributed to added sugars and ultra-processed foods. We conducted a systematic review and meta-analysis of randomized controlled trials of the effect of substituting soymilk for minimally processed cow’s milk and its modification by added sugars (sweetened versus unsweetened) on intermediate cardiometabolic outcomes as a basis for understanding the role of nutrient-dense ultra-processed plant protein foods in the transition to plant-based diets.

## Methods

### Design

We followed the Cochrane Handbook for Systematic Reviews of Interventions to conduct this systematic review and meta-analysis and reported our results by the Preferred Reporting Items for Systematic Reviews and Meta-Analysis (PRISMA) guidelines [19, 20] (Additional file 1: Table 1). To explore whether added sugars mediate any effects observed in sweetened soymilk studies, we conducted an additional systematic review and meta-analysis sub-study. This separate investigation followed the same protocol and methodology as our main study. It focused on controlled trials examining the impact of lactose in isocaloric comparisons with fructose-containing sugars (such as sucrose, high-fructose corn syrup [HFCS], or fructose) when not included in a dairy-like matrix, on all outcomes in the main study. The protocol is registered at ClinicalTrials.gov (NCT05637866).

### Data sources and search strategy

We searched MEDLINE, Embase, and the Cochrane Central Register of Controlled Trials databases through June 2024. The detailed search strategies for the main study and sub-study were based on validated search terms [21] (Additional file 1: Tables 2 and 4). Manual searches of the reference lists of included studies supplemented the systematic search.

### Study selection

The main study included randomized controlled trials in human adults with any health status. Included trials had a study duration of ≥ 3 weeks and investigated the effects of soymilk compared with cow’s milk in energy matched conditions on intermediate cardiometabolic outcomes (Additional file 1: Table 3). Trials that included other comparators that were not cow’s milk or had no viable outcome data were excluded. No restrictions were placed on language. For the sub-study, we included controlled trials involving adults of all health statuses that had a study duration of ≥ 3 weeks and investigated the effects of added sugars compared with lactose on the same intermediate cardiometabolic outcomes (Additional file 1: Table 5).

### Data extraction

A minimum of two investigators (ME, DG, SBM, AA) independently extracted relevant data from eligible studies. Extracted data included study design, sample size, sample characteristics (age, body mass index [BMI], sex, health status), intervention characteristics (soymilk volume, total sugars content, soy protein dose), control characteristics (cow’s milk volume, total sugars content, milk protein dose, milk fat content), baseline outcome levels, background diet, follow-up duration, setting, funding sources, and outcome data. The authors were contacted for missing outcome data when it was indicated that a relevant outcome was measured but not reported. Graphically presented data were extracted from figures using Plot Digitizer [22].

### Outcomes

Outcomes for the main study and sub-study included blood lipids (low-density lipoprotein cholesterol [LDL-C], high-density lipoprotein cholesterol [HDL-C], non-high-density lipoprotein cholesterol [non-HDL-C], triglycerides, and apolipoprotein B [ApoB]), glycemic control (hemoglobin A1c [HbA1c], fasting plasma glucose, 2-h postprandial glucose, fasting insulin, and plasma glucose area under the curve [PG-AUC]), blood pressure (systolic blood pressure and diastolic blood pressure), inflammation (c-reactive protein [CRP]), adiposity (body weight, BMI, body fat, and waist circumference), kidney function and structure (creatinine, creatinine clearance, glomerular filtration rate [GFR], estimated glomerular filtration rate [eGFR], albuminuria, and albumin-creatinine ratio [ACR]), uric acid, and non-alcoholic fatty liver disease (NAFLD) (intrahepatocellular lipid [IHCL], alanine transaminase [ALT], aspartate aminotransferase [AST], and fatty liver index).

Mean differences (MDs) between the intervention and control arm and respective standard errors were extracted for each trial. If these were not provided, they were derived from available data using published formulas [19]. Mean pairwise difference in change-from-baseline values were preferred over end values. When median data was provided, they were converted to mean data with corresponding variances using methods developed by McGrath et al. [23]. When no variance data was available, the standard deviation of the MDs was borrowed from a trial similar in size, participants, and nature of intervention. All disagreements were reconciled by consensus or with a senior reviewer (JLS).

### Risk of *bias* assessment

Included studies were assessed for the risk of bias independently and in duplicate by at least two investigators (ME, DG, SBM, AA) using the Cochrane Risk of Bias (ROB) 2 Tool [24]. The assessment was performed across six domains of bias (randomization process, deviations from intended interventions, missing outcome data, measurement of the outcome, selection of the reported result, and overall bias). Crossover studies were assessed for an additional domain of bias (risk of bias arising from period or carryover effects). The ROB for each domain was assessed as “low” (plausible bias unlikely to seriously alter the results), “high” (plausible bias that seriously weakens confidence in results), or “some concern” (plausible bias that raises some doubt about the results). Reviewer discrepancies were resolved by consensus or arbitration by a senior investigator (JLS).

### Statistical analysis

STATA (version 17; StataCorp LP, College Station, TX) was used for all analyses for the main study and sub-study. The principal effect measures were the mean pair-wise differences in change from baseline (or alternatively, end differences) between the intervention arm providing the soymilk and the cow’s milk comparator/control arm in each trial (significance at *P*_MD_ < 0.05). Results are reported as MDs with 95% confidence intervals (95% CI). As one of our primary research questions relates to the role of added sugars as a mediator in any observed differences between soymilk and cow’s milk, we stratified results by the presence of added sugars in the soymilk (sweetened versus unsweetened) and assessed effect modification by this variable on pooled estimates. Data were pooled using the generic inverse variance method with DerSimonian and Laird random effect models [25]. Fixed effects were used when less than five trials were available for an outcome [26]. A paired analysis was applied for crossover designs and for within-individual correlation coefficient between treatment of 0.5 as described by Elbourne et al. [27, 28].

Heterogeneity was assessed using the Cochran’s Q statistic and quantified using the *I*^2^ statistic, where *I*^2^ ≥ 50% and *P*_Q_ < 0.10 were used as evidence of substantial heterogeneity [19]. Potential sources of heterogeneity were explored using sensitivity analyses. Sensitivity analyses were done via two methods. We conducted an influence analysis by systematically removing one trial at a time and recalculating the overall effect estimate and heterogeneity. A trial was considered influential if its removal explained the substantial heterogeneity or altered the direction, magnitude, or significance of the summary estimate. To determine whether the overall summary estimates were robust to the use of an assumed correlation coefficient for crossover trials, we conducted a second sensitivity analysis by using correlation coefficients of 0.25 and 0.75. If ≥ 10 trials were available, meta-regression analyses were used to assess the significance of each subgroup categorically and when possible, continuously (significance at *P* < 0.05). A priori subgroup analyses included soy protein dose, follow-up duration, baseline outcome levels, comparator, design, age, health status, funding, and risk of bias.

If ≥ 6 trials are available [29], dose–response analyses were performed using meta-regression to assess linear (by generalized least squares trend (GLST) estimation models) and non-linear spline curve modeling (by MKSPLINE procedure) dose–response gradients (significance at *P* < 0.05).

If ≥ 10 studies were available, publication bias was assessed by inspection of contour-enhanced funnel plots and formal testing with Egger’s and Begg’s tests (significance at *P* < 0.10) [30–32]. If evidence of publication bias was suspected, the Duval and Tweedie trim-and-fill method was performed to adjust for funnel plot asymmetry by imputing missing study data and assess for small-study effects [33].

### Certainty of evidence

The Grading of Recommendations Assessment, Development, and Evaluation (GRADE) approach was used to assess the certainty of evidence. The GRADE Handbook and GRADEpro V.3.2 software were used [34, 35]. A minimum of two investigators (ME, DG, SBM) independently performed GRADE assessments for each outcome [36]. Discrepancies were resolved by consensus or arbitration by the senior author (JLS). The overall certainty of evidence was graded as either high, moderate, low, or very low. Randomized trials are initially graded as high by default and then downgraded or upgraded based on prespecified criteria. Reasons for downgrading the evidence included study limitations (risk of bias assessed by the Cochrane ROB Tool), inconsistency of results (substantial unexplained interstudy heterogeneity, *I*^2^ > 50% and *P*_Q_ < 0.10), indirectness of evidence (presence of factors that limit the generalizability of the results), imprecision (the 95% CI for effect estimates overlap with the MID for benefit or harm), and publication bias (evidence of small-study effects). The evidence was upgraded if a significant dose–response gradient was detected. We defined the importance of the magnitude of the pooled effect estimates using prespecified MIDs (Additional file 1: Table 6) with GRADE guidance [36–50] according to five levels: very large (≥ 10 MID); large (≥ 5 MID); moderate (≥ 2 MID); small important (≥ 1 MID); and trivial/unimportant (< 1 MID) effects.

## Results

### Search results

**Fig. 1:**
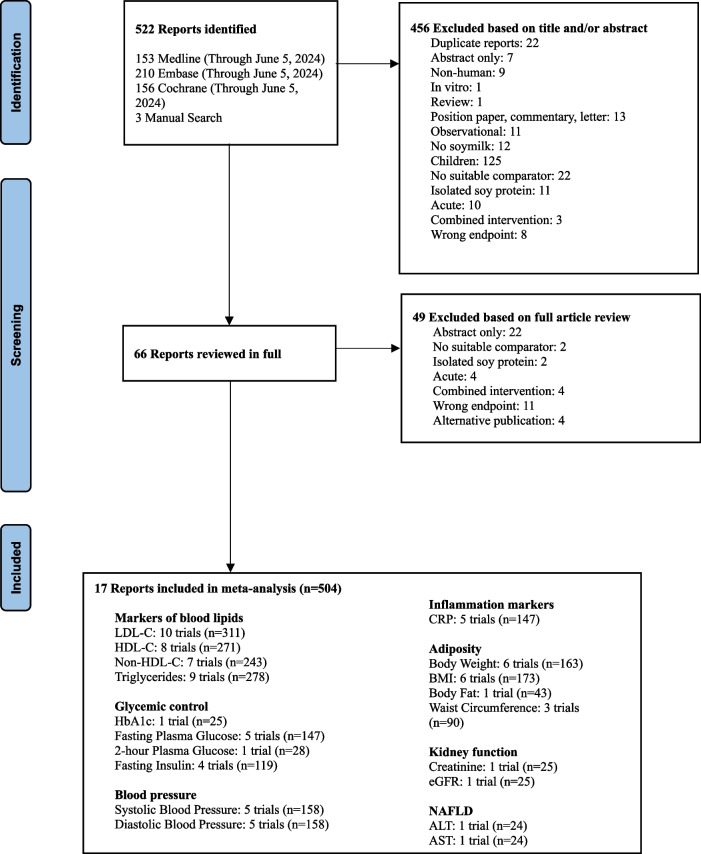
Flow of literature on the effect of substituting soymilk for cow’s milk on intermediate cardiometabolic outcomes. Exclusion criteria: duplicate, abstract only (conference abstract), non-human (animal study), in vitro, review/position paper/commentary/letter, observational (observational study), no soymilk (intervention was not soymilk), children (participants < 18 years of age), no suitable comparator (comparator was not cow’s milk), isolated soy protein (an ISP powder was given to participants), acute (follow-up of < 3 weeks), combined intervention (effects of intervention and comparator could not be isolated), wrong endpoint (no data for outcomes of interest), alternative publication (repeated data from original publication)

Figure 1 in Appendix shows the flow of the literature for the main analysis. We identified 522 reports through database and manual searches. A total of 17 reports [51–67] met the inclusion criteria and contained data for LDL (10 trials, *n* = 312), HDL-C (8 trials, *n* = 271), non-HDL-C (7 trials, *n* = 243), triglycerides (9 trials, *n* = 278), HbA1c (1 trial, *n* = 25), fasting plasma glucose (5 trials, *n* = 147), 2-h plasma glucose (1 trial, *n* = 28), fasting insulin (4 trials, *n* = 119), systolic blood pressure (5 trials, *n* = 158), diastolic blood pressure (5 trials, *n* = 158), CRP (5 trials, *n* = 147), body weight (6 trials, *n* = 163), BMI (6 trials, *n* = 173), body fat (1 trial, *n* = 43), waist circumference (3 trials, *n* = 90), creatinine (1 trial, *n* = 25), eGFR (1 trial, *n* = 25), ALT (1 trial, *n* = 24), and AST (1 trial, *n* = 24) involving 504 participants. No trials were available for ApoB, PG-AUC, creatinine clearance, eGFR, albuminuria, ACR, uric acid, IHCL, or fatty liver index.

Additional file 1: Fig. 1 shows the flow of literature for the sub-study. We identified 1010 reports through database and manual searches. After excluding 305 duplicates, a total of 705 reports were reviewed by title and abstract. No reports met the inclusion criteria and therefore no data was available for analysis.

### Trial characteristics

Table 1 shows the characteristics of the included trials. The trials were conducted in a variety of locations, with most conducted in Iran (7/17 trials, 41%), followed by the US (3/17 trials, 18%), Italy (2/17 trials, 12%), Brazil (1/17 trials, 6%), Scotland (1/17 trials, 6%), Sweden (1/17 trials, 6%), Spain (1/17 trials, 6%), and Australia (1/17 trials, 6%). All trials took place in outpatient settings (17/17, 100%). The median trial size was 25 participants (range, 7–60 participants). The median age of the participants was 48.5 years (range, 20–70 years) and the median BMI was 27.9 kg/m^2^ (range, 20–31.1 kg/m^2^). The trials included participants with hypercholesterolemia (4/17 trials, 25%), overweight or obesity (4/17 trials, 25%), type 2 diabetes (2/17 trials, 12%), hypertension (1/17 trials, 6%), rheumatoid arthritis (1/17 trials, 6%), or were healthy (3/17 trials, 18%) or post-menopausal (2/17 trials, 12%). Both trials with crossover design (10/17 trials, 59%) and parallel design (7/17 trials, 41%) were included. The intervention included sweetened (11/17 trials, 65%) and unsweetened (6/17 trials, 35%) soymilk.

The median soymilk dose was 500 mL/day (range, 240–1000 mL/day) with a median soy protein of 22 g/day (range, 2.5–70 g/day) or 6.6 g/250 mL (range, 2.6–35 g/250 mL) and median total (added) sugars of 17.2 g/day (range, 4.0–32 g/day) or 6.9 g/250 mL (range, 1–16 g/250 mL) in the sweetened soymilk. The comparators included skim (0% milk fat) (2/17 trials, 12%), low-fat (1% milk fat) (4/17 trials, 24%), reduced fat (1.5–2.5% milk fat) (7/17 trials, 41%), and whole (3% milk fat) (1/17 trials, 6%) cow’s milk. Three trials did not report the milk fat content of cow’s milk used. The median cow’s milk dose was 500 mL/day (range, 236–1000 mL/day) with a median milk protein of 24 g/day (range, 3.3–70 g/day) or 8.3 g/250 mL (range, 3.4–35 g/250 mL) and median total (lactose) sugars of 24 g/day (range, 11.5–49.2 g/day) or 12 g/250 mL (range, 10.8–12.8 g/250 mL). The median study duration was 4 weeks (range, 4–16 weeks). The trials received funding from industry (1/17 trials, 6%), agency (8/17 trials, 47%), both industry and agency (4/16 trials, 25%), or they did not report the funding source (4/17 trials, 24%).

### Risk of *bias* assessment

Additional file 1: Fig. 2 shows the ROB assessments of the included trials. Two trials were assessed as having some concerns from period or carryover effects: Bricarello et al. [53] and Steele [67]. All other trials were judged as having an overall low risk of bias. There was no evidence of serious risk of bias across the included trials.

### Outcomes

#### Markers of blood lipids

**Fig. 2:**
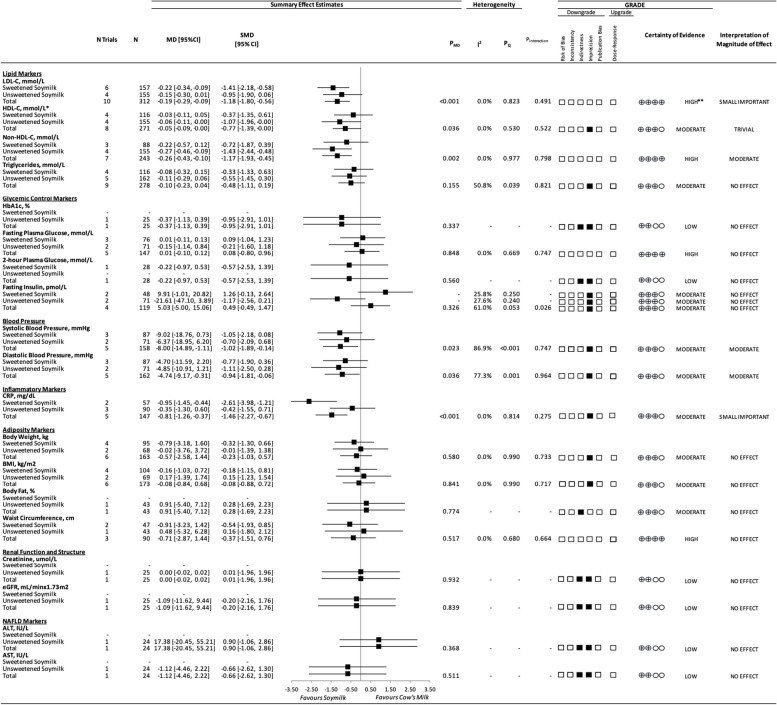
A summary plot for the effect of substituting soymilk for cow’s milk on intermediate cardiometabolic outcomes. Analyses were conducted using generic, inverse variance random-effects models (at least 5 trials available), or fixed-effects models (fewer than 5 trials available). Between-study heterogeneity was assessed by the Cochrane Q statistic, where *P*_Q_ < 0.100 was considered statistically significant, and quantified by the *I*^2^ statistic, where *I*^2^ ≥ 50% was considered evidence of substantial heterogeneity. The GRADE of randomized controlled trials are rated as “high” certainty of evidence and can be downgraded by 5 domains and upgraded by 1 domain. The white squares represent no downgrades, the filled black squares indicate a single downgrade or upgrades for each outcome, and the black square with a white “2” indicates a double downgrade for each outcome. Because all included trials were randomized or nonrandomized controlled trials, the certainty of the evidence was graded as high for all outcomes by default and then downgraded or upgraded based on prespecified criteria. Criteria for downgrades included risk of bias (downgraded if most trials were considered to be at high ROB); inconsistency (downgraded if there was substantial unexplained heterogeneity: *I*^2^ ≥ 50%; *P*_Q_ < 0.10); indirectness (downgraded if there were factors absent or present relating to the participants, interventions, or outcomes that limited the generalizability of the results); imprecision (downgraded if the 95% CI crossed the minimally important difference (MID) for harm or benefit); and publication bias (downgraded if there was evidence of publication bias based on the funnel plot asymmetry and/or significant Egger or Begg test (*P* < 0.10)), with confirmation by adjustment using the trim-and-fill analysis of Duval and Tweedie. The criteria for upgrades included a significant dose–response gradient. For the interpretation of the magnitude, we used the MIDs to assess the importance of magnitude of our point estimate using the effect size categories according to the new GRADE guidance. Then, we used the MIDs to assess the importance of the magnitude of our point estimates using the effect size categories according to the GRADE guidance as follows: a large effect (≥ 5 × MID); moderate effect (≥ 2 × MID); small important effect (≥ 1 × MID); and trivial/unimportant effect (< 1 MID). *HDL-C values reversed to show benefit. **LDL-C was not downgraded for imprecision, as the degree to which the upper 95% CI crosses the MID is not clinically meaningful. Additionally, the moderate change in non-HDL-C, with high certainty of evidence, substantiates the high certainty of the LDL-C results.

Figure 2 and Additional file 1: Figs. 3–6 show the effect of substituting soymilk for cow’s milk on markers of blood lipids. The substitution resulted in a small important reduction in LDL-C (10 trials; MD: − 0.19 mmol/L; 95% CI: − 0.29 to − 0.09 mmol/L; *P*_MD_ < 0.001; no heterogeneity: *I*^2^ = 0.0%; *P*_Q_ = 0.823), a trivial increase in HDL-C (8 trials; MD: 0.05 mmol/L; 95% CI: 0.00 to 0.09 mmol/L; *P*_MD_ = 0.036; no heterogeneity: *I*^2^ = 0.0%; *P*_Q_ = 0.053), a moderate reduction in non-HDL-C (7 trials; MD: − 0.26 mmol/L; 95% CI: − 0.43 to − 0.10 mmol/L; *P*_MD_ = 0.002; no heterogeneity: *I*^2^ = 0.0%; *P*_Q_ = 0.977), and no effect on triglycerides. There were no interactions by added sugars in soymilk for any blood lipid markers (*P* = 0.49–0.821).

#### Markers of glycemic control

Figure 2 and Additional file 1: Figs. 7–10 show the effect of substituting soymilk for cow’s milk on markers of glycemic control. The substitution had no effect on HbA1c, fasting plasma glucose, 2-h plasma glucose, or fasting insulin. There was no interaction by added sugars in soymilk for fasting plasma glucose (*P* = 0.747) but there was an interaction for fasting insulin (*P* = 0.026), where a lack of effect remained in both groups with neither the sweetened soymilk (non-significant increasing effect) nor the unsweetened soymilk (non-significant decreasing effect) showing an effect on fasting insulin. We could not assess this interaction for HbA1c or 2-h plasma glucose, as there was only one trial available for each outcome.

#### Blood pressure

Figure 2 and Additional file 1: Figs. 11 and 12 show the effect of substituting soymilk for cow’s milk on blood pressure. The substitution resulted in a moderate reduction in both systolic blood pressure (5 trials; MD: − 8.00 mmHg; 95% CI: − 14.89 to − 1.11 mmHg; *P*_MD_ = 0.023; substantial heterogeneity: *I*^2^ = 86.89%; *P*_Q_ ≤ 0.001) and diastolic blood pressure (5 trials; MD: − 4.74 mmHg; 95% CI: − 9.17 to − 0.31 mmHg; *P*_MD_ = 0.036; substantial heterogeneity: *I*^2^ = 77.3%; *P*_Q_ = 0.001). There were no interactions by added sugars in soymilk for blood pressure (*P* = 0.747 and 0.964).

#### Markers of inflammation

Figure 2 and Additional file 1: Fig. 13 show the effect of substituting soymilk for cow’s milk on markers of inflammation. The substitution resulted in a small important reduction in CRP (5 trials; MD: − 0.81 mg/dL; 95% CI: − 1.26 to − 0.37 mg/dL; *P*_MD_ = < 0.001; no heterogeneity: *I*^2^ = 0.0%; *P*_Q_ = 0.814). There was no interaction by added sugars in soymilk for CRP (*P* = 0.275).

#### Markers of adiposity

Figure 2 and Additional file 1: Figs. 14–17 show the effect of substituting soymilk for cow’s milk on markers of adiposity. The substitution had no effect on body weight, BMI, body fat, or waist circumference. There were no interactions by added sugars in soymilk for any adiposity outcome (*P* = 0.664–0.733).

#### Markers of kidney function

Figure 2 and Additional file 1: Figs. 18 and 19 show the effect of substituting soymilk for cow’s milk on markers of kidney function. The substitution had no effect on creatinine or eGFR. We could not assess the interaction by added sugars in soymilk for creatinine or eGFR, as there was only one trial available for each outcome which included soymilk without added sugars.

#### Markers of NAFLD

Figure 2 and Additional file 1: Figs. 20 and 21 show the effect of substituting soymilk for cow’s milk on markers of NAFLD. The substitution had no effect on ALT or AST. We could not assess heterogeneity or the interaction by added sugars in soymilk for ALT or AST, as there was only one trial available for each outcome which included soymilk without added sugars.

### Sensitivity analysis

Additional file 1: Figs. 22–33 present the influence analyses across all outcomes. The removal of Bricarello et al. [53] or Steele [67] each resulted in loss of significant effect for HDL-C. The removal of Onning et al. [62] or Steele [67] each resulted in a partial explanation of heterogeneity for triglycerides. The removal of Hasanpour et al. [56] explained the heterogeneity for fasting insulin. The removal of Keshavarz et al. [57] or Miraghajani et al. [59] each resulted in a loss of significant effect for systolic blood pressure and the removal of Rivas et al. [63] resulted in a partial explanation of the heterogeneity for systolic blood pressure. The removal of Hasanpour et al. [56], Keshavarz et al. [57], Miraghajani et al. [59], or Rivas et al. [63] each resulted in a loss of significant effect for diastolic blood pressure and the removal of Rivas et al. [63] resulted in a partial explanation of heterogeneity for diastolic blood pressure. The removal of Mohammad-Shahi et al. [58] resulted in loss of significant effect for CRP.

Additional file 1: Table 8 shows the sensitivity analyses for the different correlation coefficients (0.25 and 0.75) used in paired analyses of crossover trials for all outcomes. The different correlation coefficients did not alter the direction, magnitude, or significance of the effect or evidence for heterogeneity, with the following exceptions: loss of significance for the effect of the substitution on HDL-C (8 trials; MD: 0.04 mmol/L; 95% CI: − 0.10 to 0.01 mmol/L; *P*_MD_ = 0.107; *I*^2^ = 0.0%; *P*_Q_ = 0.670) with the use of 0.25 and (8 trials; MD: 0.05 mmol/L; 95% CI: − 0.10 to 0.01 mmol/L; *P*_MD_ = 0.089; *I*^2^ = 0.0%; *P*_Q_ = 0.640) with the use of 0.75.

### Subgroup analyses

Additional file 1: Figs. 34–36 present the subgroup analyses and continuous meta-regression analyses for LDL-C. Subgroup analysis was not conducted for any other outcome as there were < 10 trials included. There was no significant effect modification by health status, BMI, age, comparator, baseline LDL-C, study design, follow-up duration, funding source, dose of soy protein, or risk of bias for LDL-C. However, there were tendencies towards a greater reduction in LDL-C by point estimates in groups with certain health statuses (hypercholesterolemic and overweight/obesity), a higher baseline LDL-C, and a higher soy protein dose (> 25 g/day).

### Dose–response analyses

Additional file 1: Figs. 37–42 present linear and non-linear dose–response analyses for LDL-C, HDL-C, non-HDL-C, triglycerides, body weight, and BMI. There was no dose–response seen for the effect of substituting soymilk for cow’s milk, with the exception of a positive linear dose–response for triglycerides (*P*_linear_ = 0.038). We did not downgrade the certainty of evidence as the greater reduction in triglycerides seen at lower doses of soy protein was lost at higher doses. There were no dose–response analyses performed for the remaining outcomes because there were < 6 trials available for each.

### Publication *bias* assessment

Additional file 1: Fig. 43 presents the contour-enhanced funnel plot for assessment of publication bias for LDL-C. There was no asymmetry at the visual inspection and no evidence (Begg’s test = 0.721, Egger’s test = 0.856) of funnel plot asymmetry for LDL-C. No other publication bias analyses could be performed as there were < 10 trials available for each.

### Adverse events and acceptability

Additional file 1: Table 9 shows the reported adverse events and acceptability of study beverages. Adverse events were reported in nine trials. In one trial by Gardner et al. [55], one participant experienced a recurrence of a cancer; however, it was considered to be unrelated to the short-term consumption of the study milks. Three trials (Miraghajani et al., Hasanpour et al., and Mohammad-Shahi, et al.) [56, 58, 59] reported one to two withdrawals due to digestive difficulties related to soymilk consumption. Two trials (Sirtori et al. 1999 and 2002) [65, 66] reported one or more participants with digestive difficulties related to cow’s milk consumption. Two trials (Nourieh et al. and Keshavarz et al.) [57, 61] each reported two participant withdrawals related to digestive problems that were not specific to either study beverage. Of these, four trials indicated that most participants found the soymilk and cow’s milk acceptable and tolerable. One trial, by Onning et al. [62], incorporated a sensory evaluation of appearance, consistency, flavor, and overall impression, which showed declining scores for both types of milk over the 3-week test period.

### GRADE assessment

Additional file 1: Table 10 presents the GRADE assessment. The certainty of evidence for the effect of substituting soymilk for cow’s milk was high for LDL-C, non-HDL-C, fasting plasma glucose, and waist circumference. The certainty of evidence was moderate for HDL-C, triglycerides, fasting insulin, systolic blood pressure, diastolic blood pressure, CRP, body weight, and BMI owing to a downgrade for imprecision of the pooled effect estimates and was moderate for body fat owing to a downgrade for indirectness. The certainty of evidence was low for HbA1c, 2-h plasma glucose, creatinine, eGFR, ALT, and AST owing to downgrades for indirectness and imprecision.

## Discussion

We conducted a systematic review and meta-analysis of 17 trials that examined the effect of substituting soymilk (median dose of 22 g/day or 6.6 g/250 mL serving of soy protein per day and 17.2 g/day or 6.9 g/250 mL of total [added] sugars in the sweetened soymilk) for cow’s milk (median dose of 24 g/day or 8.3 g/250 mL of milk protein and 24 g/day or 12 g/250 mL of total sugars [lactose]) and its modification by added sugars (sweetened versus unsweetened soymilk) on 19 intermediate cardiometabolic outcomes over a median follow-up period of 4 weeks in adults of varying health status. The substitution of soymilk for cows’ milk led to moderate reductions in non-HDL-C (− 0.26 mmol/L or ~ − 7%) and systolic blood pressure (− 8.00 mmHg) and diastolic blood pressure (− 4.74 mmHg); small important reductions in LDL-C (− 0.19 mmol/L or ~ − 6%) and CRP (− 0.81 mg/L or ~ 22%); and a trivial increase in HDL-C (0.05 mmol/L or ~ 4%), with no adverse effects on other intermediate cardiometabolic outcomes. There was no meaningful interaction by added sugars in soymilk, with sweetened and unsweetened soymilk showing similar effects across outcomes. There was no dose–response relationship seen across the outcomes for which dose–response analyses were performed.

### Findings in relation to the literature

Our findings agree with previous evidence syntheses of soy. Regulatory authorities such as the United States Food and Drug Administration (FDA) and Health Canada have conducted comprehensive evaluations of the randomized controlled trials of the effect of soy protein from different sources on total-C and LDL-C, resulting in approved health claims for soy protein (based on an intake of 25 g/day of soy protein irrespective of source) for cholesterol reduction [68] and coronary heart disease risk reduction [69]. Updated systematic reviews and meta-analyses of the 46 randomized controlled trials included in the re-evaluation of the FDA health claim [70] showed reductions in LDL-C of − 3.2% [71]. This reduction has been stable since the health claim was first approved in 1999 [72] and is smaller but consistent with our findings specifically for soymilk. No increase in HDL-C, however, was detected. Previous systematic reviews and meta-analyses of randomized controlled trials of soy protein and soy isoflavones have also shown significant but smaller reductions in systolic blood pressure (1.70 mmHg) and diastolic blood pressure (− 1.27 mmHg) [73] than was found in the current analysis. These reductions in LDL-C and blood pressure are further supported by reductions in clinical events with updated pooled analyses of prospective cohort studies showing that legumes including soy are associated with reduced incidence of total cardiovascular disease and coronary heart disease [74].

Systematic reviews and meta-analyses that specifically isolated the effect of soymilk (as a single food matrix) in its intended substitution for cow’s milk are lacking. Sohouli and coworkers [75] conducted a systematic review and meta-analysis of 18 randomized controlled trials in 665 individuals of varying health status that assessed the effect of soymilk in comparison with a mix of comparators on intermediate cardiometabolic outcomes but did not isolate its substitution with cow’s milk. This synthesis showed similar improvements in LDL-C (− 0.24 mmol/L), systolic blood pressure (− 7.38 mmHg), diastolic blood pressure (− 4.36 mmHg), and CRP (− 1.07, mg/L), while also showing reductions in waist circumference and TNF-α [75]. The substitution of legumes that includes soy for various animal protein sources and more specifically legumes/nuts (the only exposure available) for dairy in syntheses of prospective cohort studies has also shown reductions in incident total cardiovascular disease and all-cause mortality [76].

Indirect evidence from dietary patterns that contain soy foods including soymilk in substitution for different animal sources of protein including cow’s milk further supports our findings. Systematic reviews and meta-analyses of randomized trials of the Portfolio diet and vegetarian and vegan dietary patterns have shown additive reductions in LDL-C, non-HDL-C, blood pressure, and CRP when soy foods including soymilk are combined with other foods that target these same intermediate risk factors with displacement of different animal sources of protein including cow’s milk [77, 78]. These reductions have also been shown to translate to reductions in clinical events with systematic reviews and meta-analyses of prospective cohort studies showing that adherence to these dietary patterns is associated with reductions in incident coronary heart disease, total cardiovascular disease, and all-cause mortality [79–81].

### Potential mechanisms of action

The potential mechanism mediating the effects of soy remains unclear. Specific components within the soy food matrix, including soy protein and phytochemicals like isoflavones [82], have been implicated. The well-established lipid-lowering effect of soy [72] may be attributed to the 7S globulin fraction of soy protein, which exerts its primary action by upregulating LDL-C receptors predominantly within the liver, thereby augmenting the clearance of LDL-C from circulation [82]. The isoflavone, fiber, fatty acids, and anti-nutrient components may also exert some mediation [83]. The reduction in blood pressure has been most linked to the soy isoflavones [83]. There is evidence that soy isoflavones may modulate the renin–angiotensin–aldosterone system (RAAS), with the capacity to inhibit the production of angiotensin II and aldosterone, thereby contributing to the regulation of blood pressure [73]. Another blood pressure lowering mechanism may involve the ability of soy isoflavones to enhance endothelial function by mitigating oxidative stress and inflammation, consequently promoting the release of the relaxing factor nitric oxide (NO) [73]. This potential mechanism of isoflavones may also explain the reductions seen in inflammation.

### Strengths and limitations

Our evidence synthesis had several strengths. First, we completed a comprehensive and reproducible systematic search and selection process of the available literature examining the effect of substituting soymilk for cow’s milk on intermediate cardiometabolic outcomes. Second, we synthesized the totality of available evidence from a large body of randomized controlled trials, which gives the greatest protection against systematic error. Third, we included an extensive and comprehensive list of outcomes to fully capture the impact of soymilk on cardiometabolic health. Fourth, we only included randomized controlled trials that compared soymilk to cow’s milk directly, to increase the specificity of our conclusion. Finally, we included a GRADE assessment to explore the certainty of available evidence.

There were also several limitations. First, we could not conduct the sub-study of the effect of lactose versus added sugars outside of a dairy-like matrix, as no eligible trials could be identified. Although this analysis is important for isolating the effect of added sugars as a mediator of any adverse effects, we did not observe any meaningful interaction by added sugars in soymilk. Second, there was serious imprecision in the pooled estimates across many of the outcomes with the 95% confidence intervals overlapping the MID in each case, with the exception of LDL-C, non-HDL-C, fasting plasma glucose, and waist circumference. The certainty of evidence for HDL-C, triglycerides, HbA1c, fasting plasma glucose, 2-h plasma glucose, fasting insulin, systolic blood pressure, diastolic blood pressure, CRP, body weight, BMI, body fat, creatinine, eGFR, ALT, and AST was downgraded for this reason. Third, there was evidence of indirectness related to insufficient trials for HbA1c, 2-h plasma glucose, creatinine, eGFR, ALT, and AST, which limits generalizability. Each outcome with data from only 1 trial was downgraded for this reason. Another source of indirectness could be the median follow-up duration of 4 weeks (range, 4–16 weeks). This time frame may be sufficient for observing certain effects, but other outcomes may require a longer period to manifest changes. Despite acknowledging this variation in response time among different outcomes, we did not further downgrade for this aspect of indirectness. Instead, we tailored our conclusions to reflect short-to-moderate term effects. Finally, although publication bias was not suspected, we were only able to make this assessment for LDL-C, as there were < 10 trials for all other outcomes.

Considering these strengths and limitations, we assessed the certainty of evidence as high for LDL-C and non-HDL-C; moderate for systolic blood pressure, diastolic blood pressure, CRP, and HDL-C; and moderate-to-low for all outcomes where significant effects were not observed.

### Implications

This work has important implications for plant protein foods in the recommended shift to more plant-based diets. Major international dietary guidelines in the US [1], Canada [3], and Europe [4–6] recommend fortified soymilk as the only suitable replacement for cow’s milk. Our findings support this recommendation showing soymilk including sweetened soymilk (up to 7 g added sugars per 250 mL) does not have any adverse effects compared with cow’s milk across 19 intermediate cardiometabolic outcomes with benefits for lipids, blood pressure, and inflammation. This evidence suggests that it may be misleading as it relates to their cardiometabolic effects to classify fortified soymilk as an ultra-processed food to be avoided while classifying cow’s milk as a minimally processed food to be encouraged (based on the WHO-endorsed NOVA classification system [10]). It also suggests that it may be misleading not to allow fortified soymilk that is sweetened with small amounts of sugars to be classified as “healthy” (based on the FDA’s new proposed definition that only permits this claim on products with added sugars ≤ 2.5 g or 5% daily value (DV) per 250 mL serving [16]). The proposed FDA criteria would prevent this claim on soymilk products designed to be iso-sweet analogs of cow’s milk (in which 5 g or 10% daily value [DV] of added sugars from sucrose in soymilk is equivalent to the 12 g of lactose in cow’s milk per 250 mL serving, as sucrose is 1.4 sweeter than lactose [17]). To prevent confusion, policy makers may want to exempt fortified soymilk from classification as an ultra-processed food and allow added sugars up to 10% DV for the definition of “healthy,” as has been proposed by the FDA for sodium and saturated fat in dairy products (including soy-based dairy alternatives) to account for accepted processing and preservation methods [16]. These policy considerations would balance the need to limit nutrient-poor energy-dense foods with the need to promote nutrient-dense foods like fortified soymilk in the shift to healthy plant-based diets.

## Conclusions

In conclusion, the evidence provides a good indication that substituting either sweetened or unsweetened soymilk for cow’s milk in adults with varying health statuses does not have the adverse effects on intermediate cardiometabolic outcomes attributed to added sugars and ultra-processed foods in the short-to-moderate term. There appear even to be advantages with small to moderate reductions in established markers of blood lipids (LDL-C, non-HDL-C) that are in line with approved health claims for cholesterol and coronary heart disease risk reduction, as well as small to moderate reductions in blood pressure and inflammation (CRP). Sources of uncertainty include imprecision and indirectness in several of the estimates. There remains a need for more well-powered randomized controlled trials of the effect of substituting soymilk for cow’s milk on less studied intermediate cardiometabolic outcomes, especially established markers of glycemic control, kidney structure and function, and NAFLD. There is also a need for trials comparing lactose versus added sugars outside of a dairy-like matrix to understand better the role of added sugars at different levels in substitution for lactose across outcomes. In the meantime, our findings support the use of fortified soymilk with up to 7 g added sugars per 250 mL as a suitable replacement for cow’s milk and suggest that its classification as ultra-processed and/or not healthy based on small amounts of added sugars may be misleading and need to be reconsidered to facilitate the recommended transition to plant-based diets.

### Supplementary Information

Additional file 1: This file contains Additional file 1 material, including the PRISMA checklist, further details on the search process, and additional results.

## Data Availability

All data generated or analyzed during this study are included in this published article and its Additional file 1: information files.

## Appendix

**Table 1:** Summary characteristics for the main analysis

	**Total (17 trials), ** ***N*** ** (range) or median (range)**	**Sweetened soymilk (11 trials), ** ***N*** ** (range) or median (range)**	**Unsweetened soymilk (6 trials), ** ***N*** ** (range) or median (range)**
**Participants**	25 (7–60)	24 (21–40)	34 (7–60)
**Health status** ^**a**^	4 HC, 4 OW/OB, 3 healthy, 2 T2D, 2 PM, 1 HTN, 1 RA	3 HC, 2 OW/OB, 2 healthy, 2 PM, 1 HTN, 1 RA	1 HC, 2 OW/OB, 1 healthy, 2 T2D
**Sex**	392 females, 112 males	229 females, 70 males	163 females, 42 males
**Age, years**	48.5 (20–70)	46 (18–70)	44.5 (20–56)
**BMI, kg/m** ^**2**^	27.9 (20–31.1)	26 (22.5–31.1)	28 (24.9–30.9)
**Setting** ^**b**^	17 OP	11 OP	6 OP
**Setting; country**	7 Iran, 3 US, 2 Italy, 1 Brazil, 1 Scotland, 1 Sweden, 1 Spain, 1 Australia	3 Iran, 3 US, 2 Italy, 1 Sweden, 1 Spain, 1 Australia	4 Iran, 1 Brazil, 1 Scotland
**Design**	10 control, 7 parallel	7 control, 4 parallel	3 control, 3 parallel
**Background diet**	12 energy neutral, 3 energy reduced, 2 protein limited	7 habitual, 2 energy reduced, 2 protein limited	5 energy neutral, 1 energy reduced
**Follow-up, weeks**	4 (4–16)	4 (4–16)	5 (4–8)
**Funding sources**	1 industry, 8 agency, 4 agency and industry, 4 undisclosed	1 industry, 4 agency, 2 agency and industry, 4 undisclosed	4 agency, 2 agency and industry
**Soymilk**			
**Added sugars**	11 yes, 6 no	11 yes	6 no
**Volume, mL**	500 (240–1000)	500 (240–1000)	450 (240–1000)
**Total sugars, g/day**	17.2 (4–32)	19.2 (4–32)	4 (0–5)
**Total sugars, g/250 mL**	6.9 (1–16)	8 (1–16)	1.3 (0–3.6)
**Protein, g/day**	22 (2.5–70)	24 (2.5–70)	14 (2.5–37)
**Protein, g/250 mL**	6.6 (2.6–35)	7.5 (2.6–35)	6.3 (2.5–37)
**Cow’s milk**			
**Added sugars**	17 no	0 yes	17 no
**Volume, mL**	500 (236–1000)	500 (240–1000)	450 (240–1000)
**Total sugars, g/day**	24 (11.5–49.2)	24 (11.9–49.2)	21.6 (11.5–48)
**Total sugars, g/250 mL**	12 (10.8–12.8)	12 (11.8–12.8)	12 (10.8–12.3)
**Protein, g/day**	24 (3.3–70)	24 (3.3–70)	15 (3.3–35)
**Protein, g/250 mL**	8.3 (3.4–35)	8.5 (3.4–35)	8.3 (3.4–8.3)
**Fat content** ^**c**^	2 skim, 3 low fat, 8 reduced fat, 1 whole, 3 undisclosed	1 skim, 2 low fat, 5 reduced fat, 1 whole, 2 undisclosed	1 skim, 1 low fat, 3 reduced fat, 1 undisclosed

^a^*OW/OB*, overweight or obesity; *PM*, post-menopausal; *HC*, hypercholesterolemic; *RA*, rheumatoid arthritis; *T2D*, type 2 diabetic; *HTN*, hypertensive. ^b^*OP*, outpatient. ^c^Skim: 0% milk fat, low fat: 1% milk fat, reduced fat: 1.5–2.5% milk fat, whole: 3% milk fat

## Abbreviations

GRADE: Grading of Recommendations, Assessment, Development, and Evaluation
non-HDL-C: Non-high-density lipoprotein cholesterol
LDL-C: Low-density lipoprotein cholesterol
CRP: C-reactive protein
HDL-C: High-density lipoprotein cholesterol
WHO: World Health Organization
US: United States
PRISMA: Preferred Reporting Items for Systematic Reviews and Meta-Analysis
HFCS: High-fructose corn syrup
BMI: Body mass index
ApoB: Apolipoprotein B
HbA1c: Hemoglobin A1c
PG-AUC: Plasma glucose area under the curve
GFR: Glomerular filtration rate
eGFR: Estimated glomerular filtration rate
ACR: Albumin-creatinine ratio
NAFLD: Non-alcoholic fatty liver disease
IHCL: Intrahepatocellular lipid
ALT: Alanine transaminase
AST: Aspartate aminotransferase
MD: Mean difference
ROB: Risk of bias
95% CI: 95% Confidence interval
GLST: Generalized least squares trend
FDA: Food and Drug Administration
TNF-a: Tumor necrosis factor alpha
RAAS: Renin-angiotensin-aldosterone system
NO: Nitric oxide
DV: Daily value
