# Supplementary material for: A systematic review and meta-analysis of randomized trials of substituting soymilk for cow’s milk and intermediate cardiometabolic outcomes: understanding the impact of dairy alternatives in the transition to plant-based diets on cardiometabolic health
Source: BMC Med. 2024 Aug 22;22:336. doi: 10.1186/s12916-024-03524-7 (PMC11340166; doi:10.1186/s12916-024-03524-7)
Supplement: Supplementary file 1 — Additional file 1: This file contains Additional file 1 material, including the PRISMA checklist, further details on the search process, and additional results. [file 12916_2024_3524_MOESM1_ESM.docx]

**A systematic review and meta-analysis of randomized trials of substituting soymilk for cow’s milk and intermediate cardiometabolic outcomes: Understanding the impact of ultra-processed dairy alternatives in the transition to plant-based diets on cardiometabolic health**

Table of Contents

[SUPPLEMENTAL FIGURES 4](#_Toc153354004)

[Supplemental Figure 1 – Flow of Literature for the sub-study analysis of the effect of lactose vs. added sugars on intermediate cardiometabolic outcomes 4](#_Toc153354005)

[Supplemental Figure 2 – Risk of Bias of trials of the effect of substituting soymilk for cow’s milk on intermediate cardiometabolic outcomes all outcome 5](#_Toc153354006)

[Supplemental Figure 3 – Forest plot of the effect of substituting soymilk for cow’s milk on LDL-C 6](#_Toc153354007)

[Supplemental Figure 4 – Forest plot of the effect of substituting soymilk for cow’s milk on HDL-C 7](#_Toc153354008)

[Supplemental Figure 5 – Forest plot of the effect of substituting soymilk for cow’s milk on Non-HDL-C 8](#_Toc153354009)

[Supplemental Figure 6 – Forest plot of the effect of substituting soymilk for cow’s milk on Triglycerides 9](#_Toc153354010)

[Supplemental Figure 7 – Forest plot of the effect of substituting soymilk for cow’s milk on HbA1c 10](#_Toc153354011)

[Supplemental Figure 8 – Forest plot of the effect of substituting soymilk for cow’s milk on Fasting Plasma Glucose 11](#_Toc153354012)

[Supplemental Figure 9 – Forest plot of the effect of substituting soymilk for cow’s milk on 2-hour Plasma Glucose 12](#_Toc153354013)

[Supplemental Figure 10 – Forest plot of the effect of substituting soymilk for cow’s milk on Fasting Insulin 13](#_Toc153354014)

[Supplemental Figure 11 – Forest plot of the effect of substituting soymilk for cow’s milk on Systolic Blood Pressure 14](#_Toc153354015)

[Supplemental Figure 12 – Forest plot of the effect of substituting soymilk for cow’s milk on Diastolic Blood Pressure 15](#_Toc153354016)

[Supplemental Figure 13 – Forest plot of the effect of substituting soymilk for cow’s milk on CRP 16](#_Toc153354017)

[Supplemental Figure 14 – Forest plot of the effect of substituting soymilk for cow’s milk on Body Weight 17](#_Toc153354018)

[Supplemental Figure 15 – Forest plot of the effect of substituting soymilk for cow’s milk on BMI 18](#_Toc153354019)

[Supplemental Figure 16 – Forest plot of the effect of substituting soymilk for cow’s milk on Body Fat 19](#_Toc153354020)

[Supplemental Figure 17 – Forest plot of the effect of substituting soymilk for cow’s milk on Waist Circumference 20](#_Toc153354021)

[Supplemental Figure 18 – Forest plot of the effect of substituting soymilk for cow’s milk on Creatinine 21](#_Toc153354022)

[Supplemental Figure 19 – Forest plot of the effect of substituting soymilk for cow’s milk on eGFR 22](#_Toc153354023)

[Supplemental Figure 20 – Forest plot of the effect of substituting soymilk for cow’s milk on ALT 23](#_Toc153354024)

[Supplemental Figure 21 – Forest plot of the effect of substituting soymilk for cow’s milk on AST 24](#_Toc153354025)

[Supplemental Figure 22 – Sensitivity analysis of the systematic removal of each trial for the effect of substituting soymilk for cow’s milk on LDL-C 25](#_Toc153354026)

[Supplemental Figure 23 – Sensitivity analysis of the systematic removal of each trial for the effect of substituting soymilk for cow’s milk on HDL-C 26](#_Toc153354027)

[Supplemental Figure 24 – Sensitivity analysis of the systematic removal of each trial for the effect of substituting soymilk for cow’s milk on Non-HDL-C 27](#_Toc153354028)

[Supplemental Figure 25 – Sensitivity analysis of the systematic removal of each trial for the effect of substituting soymilk for cow’s milk on Triglycerides 28](#_Toc153354029)

[Supplemental Figure 26 – Sensitivity analysis of the systematic removal of each trial for the effect of substituting soymilk for cow’s milk on Fasting Plasma Glucose 29](#_Toc153354030)

[Supplemental Figure 27 – Sensitivity analysis of the systematic removal of each trial for the effect of substituting soymilk for cow’s milk on Fasting Insulin 30](#_Toc153354031)

[Supplemental Figure 28 – Sensitivity analysis of the systematic removal of each trial for the effect of substituting soymilk for cow’s milk on Systolic Blood Pressure 31](#_Toc153354032)

[Supplemental Figure 29 – Sensitivity analysis of the systematic removal of each trial for the effect of substituting soymilk for cow’s milk on Diastolic Blood Pressure 32](#_Toc153354033)

[Supplemental Figure 30 - Sensitivity analysis of the systematic removal of each trial for the effect of substituting soymilk for cow’s milk on CRP 33](#_Toc153354034)

[Supplemental Figure 31 – Sensitivity analysis of the systematic removal of each trial for the effect of substituting soymilk for cow’s milk on Body Weight 34](#_Toc153354035)

[Supplemental Figure 32 – Sensitivity analysis of the systematic removal of each trial for the effect of substituting soymilk for cow’s milk on BMI 35](#_Toc153354036)

[Supplemental Figure 33 – Sensitivity analysis of the systematic removal of each trial for the effect of substituting soymilk for cow’s milk on Waist Circumference 36](#_Toc153354037)

[Supplemental Figure 34 – Subgroup analysis for the effect of substituting soymilk for cow’s milk on LDL-C 37](#_Toc153354038)

[Supplemental Figure 35 – Risk of bias (using the Cochrane collaboration tool) subgroup analysis for the effect of substituting soymilk for cow’s milk on LDL-C 39](#_Toc153354039)

[Supplemental Figure 36 – Continuous meta-regression analysis for the effect of substituting soymilk for cow’s milk on LDL-C 40](#_Toc153354040)

[Supplemental Figure 37 – Dose response for the effect of substituting soymilk for cow’s milk on LDL-C 41](#_Toc153354041)

[Supplemental Figure 38 – Dose response for the effect of substituting soymilk for cow’s milk on HDL-C 42](#_Toc153354042)

[Supplemental Figure 39 – Dose response for the effect of substituting soymilk for cow’s milk on Non-HDL-C 43](#_Toc153354043)

[Supplemental Figure 40 – Dose response for the effect of substituting soymilk for cow’s milk on Triglycerides 44](#_Toc153354044)

[Supplemental Figure 41 – Dose response for the effect of substituting soymilk for cow’s milk on Body Weight 45](#_Toc153354045)

[Supplemental Figure 42 – Dose response for the effect of substituting soymilk for cow’s milk on BMI 45](#_Toc153354046)

[Supplemental Figure 43 – Publication bias funnel plot for the effect of substituting soymilk for cow’s milk on LDL-C 47](#_Toc153354047)

[SUPPLEMENTAL TABLES 48](#_Toc153354048)

[Supplemental Table 1 – PRISMA Checklist 49](#_Toc153354049)

[Supplemental Table 2 – Search strategy for the effect of substituting soymilk for cow’s milk on intermediate cardiometabolic outcomes 52](#_Toc153354050)

[Supplemental Table 3 – PICOTS table of the search strategy for the effect of substituting soymilk for cow’s milk on intermediate cardiometabolic outcomes 53](#_Toc153354051)

[Supplemental Table 4 – Search strategy for sub-study analysis on the effect of lactose vs. added sugars on intermediate cardiometabolic outcomes 54](#_Toc153354052)

[Supplemental Table 5 – PICOTS table of the search strategy for the sub-study analysis on the effect of lactose vs. added sugars on intermediate cardiometabolic outcomes 55](#_Toc153354053)

[Supplemental Table 6 – Minimum Important Differences 56](#_Toc153354054)

[Supplemental Table 7 - Trial characteristics for the main analysis 58](#_Toc153354055)

[Supplemental Table 8 - Sensitivity analyses of the use of correlation coefficient of 0.25 and 0.75 for paired analysis in the analysis of the effect of substituting soymilk for cow’s milk on intermediate cardiometabolic outcomes 59](#_Toc153354056)

[Supplemental Table 9 - Adverse Events and Assessment of Acceptability 60](#_Toc153354057)

[Supplemental Table 10 - GRADE certainty of evidence assessment for the effect of substituting soymilk for cow’s milk on intermediate cardiometabolic outcomes 61](#_Toc153354058)

# SUPPLEMENTAL FIGURES

## Supplemental Figure 1 – Flow of Literature for the sub-study analysis of the effect of lactose vs. added sugars on intermediate cardiometabolic outcomes

**1010 Excluded based on title and/or abstract**

Duplicate reports: 305

Abstract only: 13

Non-human: 10

In vitro: 11

Review: 10

Position paper, commentary, letter: 20

Observational: 15

No lactose: 107

Children: 148

No suitable comparator: 42

Acute: 3

Wrong endpoint: 13

Case study: 3

Lactose intolerance: 69

Cow’s Milk: 25

Probiotic: 26

**1010 Reports identified**

217 Medline (1946-May 5, 2023)

441 Embase (1947-May 5, 2023)

352 Cochrane (Through May 5, 2023)

0 Manual Search

**Identification**

**Screening**

**0 Reports reviewed in full**

## Supplemental Figure 2 – Risk of Bias of trials of the effect of substituting soymilk for cow’s milk on intermediate cardiometabolic outcomes all outcome


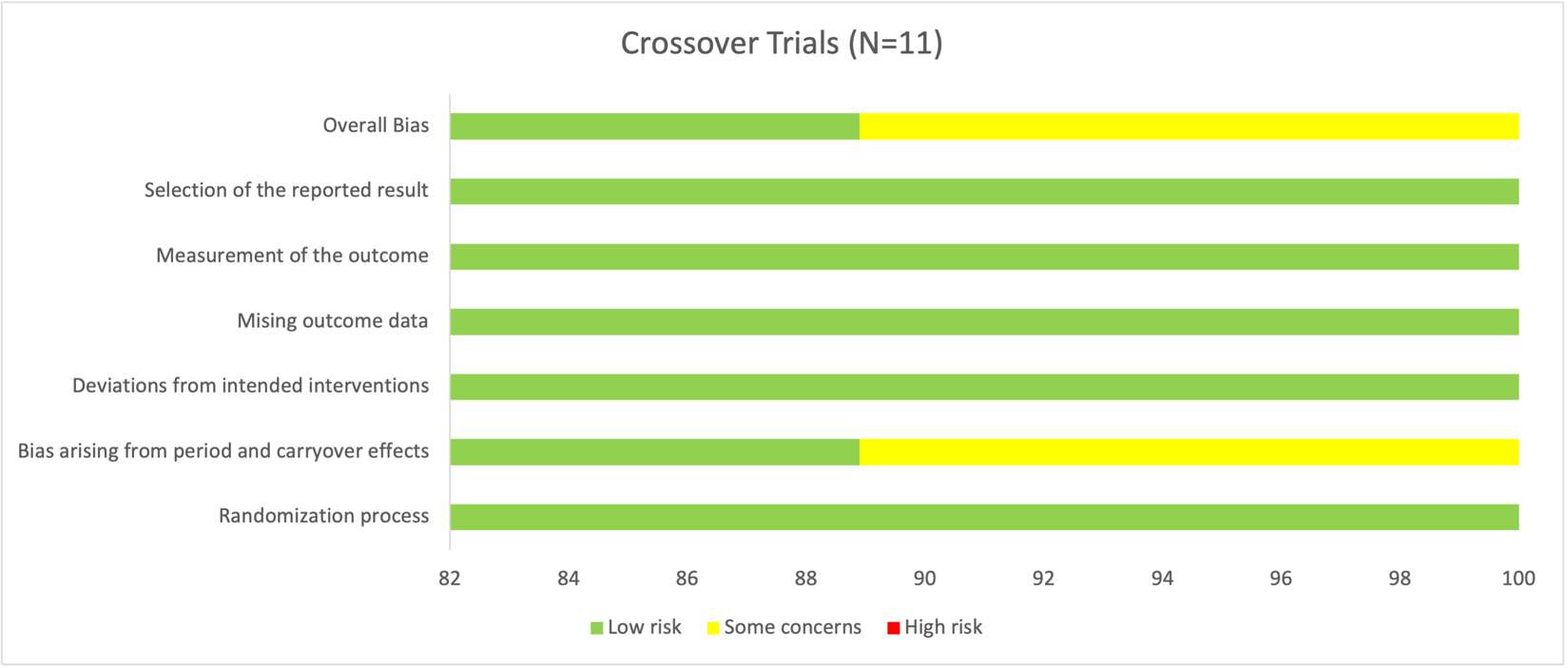


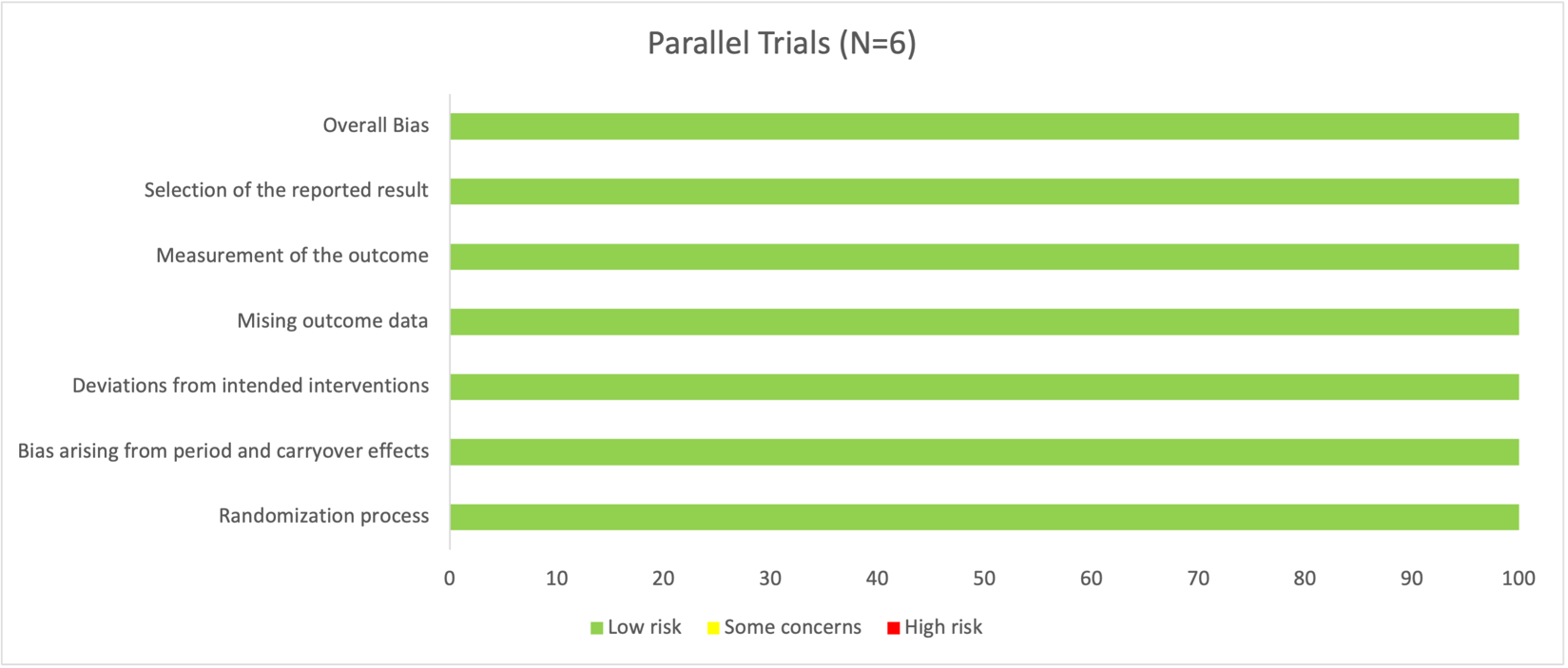


Colored bars represent the proportion of trials assessed as low (green), some concerns (yellow), or high (red) risk of bias for the six domains of bias above according to criteria set by the Cochrane Risk of Bias tool 2 in the 17 included controlled trials.

## Supplemental Figure 3 – Forest plot of the effect of substituting soymilk for cow’s milk on LDL-C


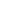

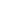

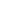


The total pooled effect estimate is represented by the green diamond. Data are expressed as MDs with 95% CIs using the generic inverse variance method modelled by random effects (DerSimonian Laird). Heterogeneity was assessed using the Cochran’s Q statistic and quantified using the I^2^ statistic, where p<0.100 and I^2^ ≥50.00% were used as evidence of significant substantial heterogeneity. Risk of Bias Legend: (H) High Risk; (L) Low Risk; (S) Some Concerns. The letters represent the following risk of bias domains: A, random sequence generation (selection bias); B, risk of bias arising from period or carryover effects; C, deviations from intended interventions; D, incomplete outcome data (attrition bias); E, measurement of the outcome; F, selective reporting (reporting bias); and G, overall bias. Risk of bias arising from period and carryover effects was only applicable to crossover trials. CI, confidence interval. *SE(MD) for Sirtori 2002 was borrowed from Nourieh 2012 due to data unavailability.

## Supplemental Figure 4 – Forest plot of the effect of substituting soymilk for cow’s milk on HDL-C

**
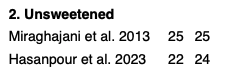
**
The total pooled effect estimate is represented by the green diamond. Data are expressed as MDs with 95% CIs using the generic inverse variance method modelled by random effects (DerSimonian Laird). Heterogeneity was assessed using the Cochran’s Q statistic and quantified using the I^2^ statistic, where p<0.100 and I^2^ ≥50.00% were used as evidence of significant substantial heterogeneity. Risk of Bias Legend: (H) High Risk; (L) Low Risk; (S) Some Concerns. The letters represent the following risk of bias domains: A, random sequence generation (selection bias); B, risk of bias arising from period or carryover effects; C, deviations from intended interventions; D, incomplete outcome data (attrition bias); E, measurement of the outcome; F, selective reporting (reporting bias); and G, overall bias. Risk of bias arising from period and carryover effects was only applicable to crossover trials. CI, confidence interval.


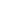

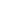

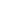

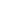


## Supplemental Figure 5 – Forest plot of the effect of substituting soymilk for cow’s milk on Non-HDL-C

**
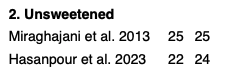
**

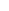

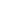

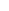


The total pooled effect estimate is represented by the green diamond. Data are expressed as MDs with 95% CIs using the generic inverse variance method modelled by random effects (DerSimonian Laird). Heterogeneity was assessed using the Cochran’s Q statistic and quantified using the I^2^ statistic, where p<0.100 and I^2^ ≥50.00% were used as evidence of significant substantial heterogeneity. Risk of Bias Legend: (H) High Risk; (L) Low Risk; (S) Some Concerns. The letters represent the following risk of bias domains: A, random sequence generation (selection bias); B, risk of bias arising from period or carryover effects; C, deviations from intended interventions; D, incomplete outcome data (attrition bias); E, measurement of the outcome; F, selective reporting (reporting bias); and G, overall bias. Risk of bias arising from period and carryover effects was only applicable to crossover trials. CI, confidence interval. *SE(MD) for Onning 1998 was borrowed from Beavers 2010 due to data unavailability.

## Supplemental Figure 6 – Forest plot of the effect of substituting soymilk for cow’s milk on Triglycerides

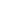

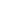

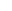

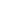


The total pooled effect estimate is represented by the green diamond. Data are expressed as MDs with 95% CIs using the generic inverse variance method modelled by random effects (DerSimonian Laird). Heterogeneity was assessed using the Cochran’s Q statistic and quantified using the I^2^ statistic, where p<0.100 and I^2^ ≥50.00% were used as evidence of significant substantial heterogeneity. Risk of Bias Legend: (H) High Risk; (L) Low Risk; (S) Some Concerns. The letters represent the following risk of bias domains: A, random sequence generation (selection bias); B, risk of bias arising from period or carryover effects; C, deviations from intended interventions; D, incomplete outcome data (attrition bias); E, measurement of the outcome; F, selective reporting (reporting bias); and G, overall bias. Risk of bias arising from period and carryover effects was only applicable to crossover trials. CI, confidence interval.

## Supplemental Figure 7 – Forest plot of the effect of substituting soymilk for cow’s milk on HbA1c

The total pooled effect estimate is represented by the green diamond. Data are expressed as MDs with 95% CIs using the generic inverse variance method modelled by random effects (DerSimonian Laird). Heterogeneity was assessed using the Cochran’s Q statistic and quantified using the I^2^ statistic, where p<0.100 and I^2^ ≥50.00% were used as evidence of significant substantial heterogeneity. Risk of Bias Legend: (H) High Risk; (L) Low Risk; (S) Some Concerns. The letters represent the following risk of bias domains: A, random sequence generation (selection bias); B, risk of bias arising from period or carryover effects; C, deviations from intended interventions; D, incomplete outcome data (attrition bias); E, measurement of the outcome; F, selective reporting (reporting bias); and G, overall bias. Risk of bias arising from period and carryover effects was only applicable to crossover trials. CI, confidence interval.

## Supplemental Figure 8 – Forest plot of the effect of substituting soymilk for cow’s milk on Fasting Plasma Glucose

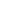

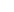


The total pooled effect estimate is represented by the green diamond. Data are expressed as MDs with 95% CIs using the generic inverse variance method modelled by random effects (DerSimonian Laird). Heterogeneity was assessed using the Cochran’s Q statistic and quantified using the I^2^ statistic, where p<0.100 and I^2^ ≥50.00% were used as evidence of significant substantial heterogeneity. Risk of Bias Legend: (H) High Risk; (L) Low Risk; (S) Some Concerns. The letters represent the following risk of bias domains: A, random sequence generation (selection bias); B, risk of bias arising from period or carryover effects; C, deviations from intended interventions; D, incomplete outcome data (attrition bias); E, measurement of the outcome; F, selective reporting (reporting bias); and G, overall bias. Risk of bias arising from period and carryover effects was only applicable to crossover trials. CI, confidence interval.

## Supplemental Figure 9 – Forest plot of the effect of substituting soymilk for cow’s milk on 2-hour Plasma Glucose


The total pooled effect estimate is represented by the green diamond. Data are expressed as MDs with 95% CIs using the generic inverse variance method modelled by random effects (DerSimonian Laird). Heterogeneity was assessed using the Cochran’s Q statistic and quantified using the I^2^ statistic, where p<0.100 and I^2^ ≥50.00% were used as evidence of significant substantial heterogeneity. Risk of Bias Legend: (H) High Risk; (L) Low Risk; (S) Some Concerns. The letters represent the following risk of bias domains: A, random sequence generation (selection bias); B, risk of bias arising from period or carryover effects; C, deviations from intended interventions; D, incomplete outcome data (attrition bias); E, measurement of the outcome; F, selective reporting (reporting bias); and G, overall bias. Risk of bias arising from period and carryover effects was only applicable to crossover trials. CI, confidence interval.

## Supplemental Figure 10 – Forest plot of the effect of substituting soymilk for cow’s milk on Fasting Insulin

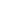

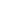


The total pooled effect estimate is represented by the green diamond. Data are expressed as MDs with 95% CIs using the generic inverse variance method modelled by random effects (DerSimonian Laird). Heterogeneity was assessed using the Cochran’s Q statistic and quantified using the I^2^ statistic, where p<0.100 and I^2^ ≥50.00% were used as evidence of significant substantial heterogeneity. Risk of Bias Legend: (H) High Risk; (L) Low Risk; (S) Some Concerns. The letters represent the following risk of bias domains: A, random sequence generation (selection bias); B, risk of bias arising from period or carryover effects; C, deviations from intended interventions; D, incomplete outcome data (attrition bias); E, measurement of the outcome; F, selective reporting (reporting bias); and G, overall bias. Risk of bias arising from period and carryover effects was only applicable to crossover trials. CI, confidence interval.

## Supplemental Figure 11 – Forest plot of the effect of substituting soymilk for cow’s milk on Systolic Blood Pressure

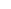

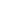


The total pooled effect estimate is represented by the green diamond. Data are expressed as MDs with 95% CIs using the generic inverse variance method modelled by random effects (DerSimonian Laird). Heterogeneity was assessed using the Cochran’s Q statistic and quantified using the I^2^ statistic, where p<0.100 and I^2^ ≥50.00% were used as evidence of significant substantial heterogeneity. Risk of Bias Legend: (H) High Risk; (L) Low Risk; (S) Some Concerns. The letters represent the following risk of bias domains: A, random sequence generation (selection bias); B, risk of bias arising from period or carryover effects; C, deviations from intended interventions; D, incomplete outcome data (attrition bias); E, measurement of the outcome; F, selective reporting (reporting bias); and G, overall bias. Risk of bias arising from period and carryover effects was only applicable to crossover trials. CI, confidence interval. *Keshavarz et al. 2012 reported implausible baseline and end values for systolic blood pressure, therefore we adjusted the decimal point placement to align the data with the other trials.

## Supplemental Figure 12 – Forest plot of the effect of substituting soymilk for cow’s milk on Diastolic Blood Pressure

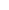

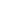


The total pooled effect estimate is represented by the green diamond. Data are expressed as MDs with 95% CIs using the generic inverse variance method modelled by random effects (DerSimonian Laird). Heterogeneity was assessed using the Cochran’s Q statistic and quantified using the I^2^ statistic, where p<0.100 and I^2^ ≥50.00% were used as evidence of significant substantial heterogeneity. Risk of Bias Legend: (H) High Risk; (L) Low Risk; (S) Some Concerns. The letters represent the following risk of bias domains: A, random sequence generation (selection bias); B, risk of bias arising from period or carryover effects; C, deviations from intended interventions; D, incomplete outcome data (attrition bias); E, measurement of the outcome; F, selective reporting (reporting bias); and G, overall bias. Risk of bias arising from period and carryover effects was only applicable to crossover trials. CI, confidence interval. *Keshavarz et al. 2012 reported implausible baseline and end values for diastolic blood pressure, therefore we adjusted the decimal point placement to align the data with the other trials.

## Supplemental Figure 13 – Forest plot of the effect of substituting soymilk for cow’s milk on CRP


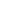

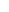


The total pooled effect estimate is represented by the green diamond. Data are expressed as MDs with 95% CIs using the generic inverse variance method modelled by random effects (DerSimonian Laird). Heterogeneity was assessed using the Cochran’s Q statistic and quantified using the I^2^ statistic, where p<0.100 and I^2^ ≥50.00% were used as evidence of significant substantial heterogeneity. Risk of Bias Legend: (H) High Risk; (L) Low Risk; (S) Some Concerns. The letters represent the following risk of bias domains: A, random sequence generation (selection bias); B, risk of bias arising from period or carryover effects; C, deviations from intended interventions; D, incomplete outcome data (attrition bias); E, measurement of the outcome; F, selective reporting (reporting bias); and G, overall bias. Risk of bias arising from period and carryover effects was only applicable to crossover trials. CI, confidence interval. *Faghih et al. 2011 reported CRP in units of ng/mL, which appeared to be incorrect, therefore we interpreted the data in units of mg/L. **Miraghajani et al. 2012 reported CRP adjusted for carbohydrate intake and sex. ***Mohammad-Shahi et al. 2016 reported CRP adjusted for weight change.

## Supplemental Figure 14 – Forest plot of the effect of substituting soymilk for cow’s milk on Body Weight

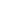

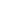

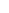


The total pooled effect estimate is represented by the green diamond. Data are expressed as MDs with 95% CIs using the generic inverse variance method modelled by random effects (DerSimonian Laird). Heterogeneity was assessed using the Cochran’s Q statistic and quantified using the I^2^ statistic, where p<0.100 and I^2^ ≥50.00% were used as evidence of significant substantial heterogeneity. Risk of Bias Legend: (H) High Risk; (L) Low Risk; (S) Some Concerns. The letters represent the following risk of bias domains: A, random sequence generation (selection bias); B, risk of bias arising from period or carryover effects; C, deviations from intended interventions; D, incomplete outcome data (attrition bias); E, measurement of the outcome; F, selective reporting (reporting bias); and G, overall bias. Risk of bias arising from period and carryover effects was only applicable to crossover trials. CI, confidence interval. *Faghih et al. 2011 reported implausible SEs for body weight, therefore we interpreted this as SD, instead.

## Supplemental Figure 15 – Forest plot of the effect of substituting soymilk for cow’s milk on BMI

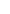

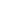

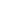


The total pooled effect estimate is represented by the green diamond. Data are expressed as MDs with 95% CIs using the generic inverse variance method modelled by random effects (DerSimonian Laird). Heterogeneity was assessed using the Cochran’s Q statistic and quantified using the I^2^ statistic, where p<0.100 and I^2^ ≥50.00% were used as evidence of significant substantial heterogeneity. Risk of Bias Legend: (H) High Risk; (L) Low Risk; (S) Some Concerns. The letters represent the following risk of bias domains: A, random sequence generation (selection bias); B, risk of bias arising from period or carryover effects; C, deviations from intended interventions; D, incomplete outcome data (attrition bias); E, measurement of the outcome; F, selective reporting (reporting bias); and G, overall bias. Risk of bias arising from period and carryover effects was only applicable to crossover trials. CI, confidence interval. *Faghih et al. 2011 reported implausible SEs for BMI, therefore we interpreted this as SD, instead.

## Supplemental Figure 16 – Forest plot of the effect of substituting soymilk for cow’s milk on Body Fat

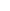


The total pooled effect estimate is represented by the green diamond. Data are expressed as MDs with 95% CIs using the generic inverse variance method modelled by random effects (DerSimonian Laird). Heterogeneity was assessed using the Cochran’s Q statistic and quantified using the I^2^ statistic, where p<0.100 and I^2^ ≥50.00% were used as evidence of significant substantial heterogeneity. Risk of Bias Legend: (H) High Risk; (L) Low Risk; (S) Some Concerns. The letters represent the following risk of bias domains: A, random sequence generation (selection bias); B, risk of bias arising from period or carryover effects; C, deviations from intended interventions; D, incomplete outcome data (attrition bias); E, measurement of the outcome; F, selective reporting (reporting bias); and G, overall bias. Risk of bias arising from period and carryover effects was only applicable to crossover trials. CI, confidence interval. *Faghih et al. 2011 reported implausible SEs for body fat, therefore we interpreted this as SD, instead.

## Supplemental Figure 17 – Forest plot of the effect of substituting soymilk for cow’s milk on Waist Circumference

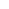


The total pooled effect estimate is represented by the green diamond. Data are expressed as MDs with 95% CIs using the generic inverse variance method modelled by random effects (DerSimonian Laird). Heterogeneity was assessed using the Cochran’s Q statistic and quantified using the I^2^ statistic, where p<0.100 and I^2^ ≥50.00% were used as evidence of significant substantial heterogeneity. Risk of Bias Legend: (H) High Risk; (L) Low Risk; (S) Some Concerns. The letters represent the following risk of bias domains: A, random sequence generation (selection bias); B, risk of bias arising from period or carryover effects; C, deviations from intended interventions; D, incomplete outcome data (attrition bias); E, measurement of the outcome; F, selective reporting (reporting bias); and G, overall bias. Risk of bias arising from period and carryover effects was only applicable to crossover trials. CI, confidence interval. *Faghih et al. 2011 reported implausible SEs for waist circumference, therefore we interpreted this as SD, instead.

## Supplemental Figure 18 – Forest plot of the effect of substituting soymilk for cow’s milk on Creatinine

The total pooled effect estimate is represented by the green diamond. Data are expressed as MDs with 95% CIs using the generic inverse variance method modelled by random effects (DerSimonian Laird). Heterogeneity was assessed using the Cochran’s Q statistic and quantified using the I^2^ statistic, where p<0.100 and I^2^ ≥50.00% were used as evidence of significant substantial heterogeneity. Risk of Bias Legend: (H) High Risk; (L) Low Risk; (S) Some Concerns. The letters represent the following risk of bias domains: A, random sequence generation (selection bias); B, risk of bias arising from period or carryover effects; C, deviations from intended interventions; D, incomplete outcome data (attrition bias); E, measurement of the outcome; F, selective reporting (reporting bias); and G, overall bias. Risk of bias arising from period and carryover effects was only applicable to crossover trials. CI, confidence interval.

## Supplemental Figure 19 – Forest plot of the effect of substituting soymilk for cow’s milk on eGFR

The total pooled effect estimate is represented by the green diamond. Data are expressed as MDs with 95% CIs using the generic inverse variance method modelled by random effects (DerSimonian Laird). Heterogeneity was assessed using the Cochran’s Q statistic and quantified using the I^2^ statistic, where p<0.100 and I^2^ ≥50.00% were used as evidence of significant substantial heterogeneity. Risk of Bias Legend: (H) High Risk; (L) Low Risk; (S) Some Concerns. The letters represent the following risk of bias domains: A, random sequence generation (selection bias); B, risk of bias arising from period or carryover effects; C, deviations from intended interventions; D, incomplete outcome data (attrition bias); E, measurement of the outcome; F, selective reporting (reporting bias); and G, overall bias. Risk of bias arising from period and carryover effects was only applicable to crossover trials. CI, confidence interval.

## Supplemental Figure 20 – Forest plot of the effect of substituting soymilk for cow’s milk on ALT

The total pooled effect estimate is represented by the green diamond. Data are expressed as MDs with 95% CIs using the generic inverse variance method modelled by random effects (DerSimonian Laird). Heterogeneity was assessed using the Cochran’s Q statistic and quantified using the I^2^ statistic, where p<0.100 and I^2^ ≥50.00% were used as evidence of significant substantial heterogeneity. Risk of Bias Legend: (H) High Risk; (L) Low Risk; (S) Some Concerns. The letters represent the following risk of bias domains: A, random sequence generation (selection bias); B, risk of bias arising from period or carryover effects; C, deviations from intended interventions; D, incomplete outcome data (attrition bias); E, measurement of the outcome; F, selective reporting (reporting bias); and G, overall bias. Risk of bias arising from period and carryover effects was only applicable to crossover trials. CI, confidence interval.

## Supplemental Figure 21 – Forest plot of the effect of substituting soymilk for cow’s milk on AST

The total pooled effect estimate is represented by the green diamond. Data are expressed as MDs with 95% CIs using the generic inverse variance method modelled by random effects (DerSimonian Laird). Heterogeneity was assessed using the Cochran’s Q statistic and quantified using the I^2^ statistic, where p<0.100 and I^2^ ≥50.00% were used as evidence of significant substantial heterogeneity. Risk of Bias Legend: (H) High Risk; (L) Low Risk; (S) Some Concerns. The letters represent the following risk of bias domains: A, random sequence generation (selection bias); B, risk of bias arising from period or carryover effects; C, deviations from intended interventions; D, incomplete outcome data (attrition bias); E, measurement of the outcome; F, selective reporting (reporting bias); and G, overall bias. Risk of bias arising from period and carryover effects was only applicable to crossover trials. CI, confidence interval.


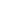

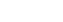


## Supplemental Figure 22 – Sensitivity analysis of the systematic removal of each trial for the effect of substituting soymilk for cow’s milk on LDL-C

## Supplemental Figure 23 – Sensitivity analysis of the systematic removal of each trial for the effect of substituting soymilk for cow’s milk on HDL-C


## Supplemental Figure 24 – Sensitivity analysis of the systematic removal of each trial for the effect of substituting soymilk for cow’s milk on Non-HDL-C


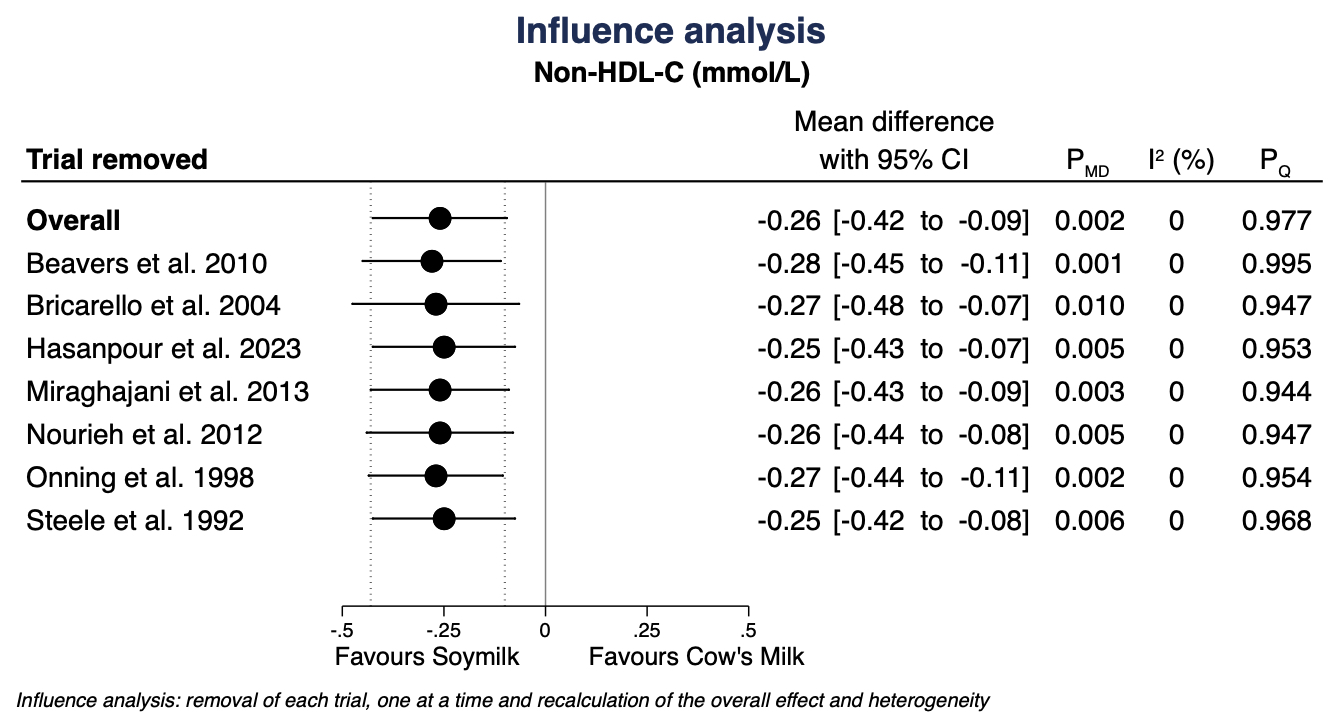


## Supplemental Figure 25 – Sensitivity analysis of the systematic removal of each trial for the effect of substituting soymilk for cow’s milk on Triglycerides

## Supplemental Figure 26 – Sensitivity analysis of the systematic removal of each trial for the effect of substituting soymilk for cow’s milk on Fasting Plasma Glucose

## Supplemental Figure 27 – Sensitivity analysis of the systematic removal of each trial for the effect of substituting soymilk for cow’s milk on Fasting Insulin

## Supplemental Figure 28 – Sensitivity analysis of the systematic removal of each trial for the effect of substituting soymilk for cow’s milk on Systolic Blood Pressure

## Supplemental Figure 29 – Sensitivity analysis of the systematic removal of each trial for the effect of substituting soymilk for cow’s milk on Diastolic Blood Pressure

## Supplemental Figure 30 - Sensitivity analysis of the systematic removal of each trial for the effect of substituting soymilk for cow’s milk on CRP


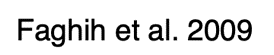


## Supplemental Figure 31 – Sensitivity analysis of the systematic removal of each trial for the effect of substituting soymilk for cow’s milk on Body Weight

## Supplemental Figure 32 – Sensitivity analysis of the systematic removal of each trial for the effect of substituting soymilk for cow’s milk on BMI

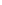


## Supplemental Figure 33 – Sensitivity analysis of the systematic removal of each trial for the effect of substituting soymilk for cow’s milk on Waist Circumference

## Supplemental Figure 34 – Subgroup analysis for the effect of substituting soymilk for cow’s milk on LDL-C

The green diamond represents the pooled estimate for the overall primary analysis. Within subgroup mean differences are the pooled effect estimates represented by a red circle. 95% confidence intervals are represented by the line through the circle. Data are expressed as mean differences with 95% confidence intervals using the generic inverse-variance method and random effects DerSimonian-Laird model. Inter‐study heterogeneity was assessed using the Cochran’s Q statistic and quantified using the I^2^ statistic, with significance set at p<0.100 and I^2^≥50% considered to be evidence of substantial heterogeneity. p<0.050 indicates that the effect size differed between levels of the subgroup. * HC: hypercholesterolemic; OW/OB: overweight or obesity; PM: post-menopausal; T2D: type 2 diabetic

## Supplemental Figure 35 – Risk of bias (using the Cochrane collaboration tool) subgroup analysis for the effect of substituting soymilk for cow’s milk on LDL-C

The green diamond represents the pooled estimate for the overall primary analysis. Within subgroup mean differences are the pooled effect estimates represented by a red circle. 95% confidence intervals are represented by the line through the circle. Data are expressed as mean differences with 95% confidence intervals using the generic inverse-variance method and random effects DerSimonian-Laird model. Inter‐study heterogeneity was assessed using the Cochran’s Q statistic and quantified using the I^2^ statistic, with significance set at p<0.100 and I^2^≥50% considered to be evidence of substantial heterogeneity. p<0.050 indicates that the effect size differed between levels of the subgroup.

## Supplemental Figure 36 – Continuous meta-regression analysis for the effect of substituting soymilk for cow’s milk on LDL-C

ß–coefficients were estimated using continuous meta-regression analysis. A positive ß-coefficient implies an increase in LDL-C as the subgroup variable increases, and a negative ß-coefficient implies a decrease in LDL-C. Residual I^2^ reports inter-study heterogeneity not explained by the subgroup and was estimated using the Cochran’s Q statistic.

## Supplemental Figure 37 – Dose response for the effect of substituting soymilk for cow’s milk on LDL-C

Individual trials are represented by the circles, with the weight of the comparison in the analysis represented by the size of the circle. The solid, orange line represents the linear dose-response modelled by random effect with restricted maximum likelihood methods. The solid black and dashed lines represent the non-linear dose-response and 95% CIs, respectively, modelled with restricted cubic splines.

## Supplemental Figure 38 – Dose response for the effect of substituting soymilk for cow’s milk on HDL-C

Individual trials are represented by the circles, with the weight of the comparison in the analysis represented by the size of the circle. The solid, orange line represents the linear dose-response modelled by random effect with restricted maximum likelihood methods. The solid black and dashed lines represent the non-linear dose-response and 95% CIs, respectively, modelled with restricted cubic splines.

## Supplemental Figure 39 – Dose response for the effect of substituting soymilk for cow’s milk on Non-HDL-C

Individual trials are represented by the circles, with the weight of the comparison in the analysis represented by the size of the circle. The solid, orange line represents the linear dose-response modelled by random effect with restricted maximum likelihood methods. The solid black and dashed lines represent the non-linear dose-response and 95% CIs, respectively, modelled with restricted cubic splines.

## Supplemental Figure 40 – Dose response for the effect of substituting soymilk for cow’s milk on Triglycerides

Individual trials are represented by the circles, with the weight of the comparison in the analysis represented by the size of the circle. The solid, orange line represents the linear dose-response modelled by random effect with restricted maximum likelihood methods. The solid black and dashed lines represent the non-linear dose-response and 95% CIs, respectively, modelled with restricted cubic splines.

## Supplemental Figure 41 – Dose response for the effect of substituting soymilk for cow’s milk on Body Weight

Individual trials are represented by the circles, with the weight of the comparison in the analysis represented by the size of the circle. The solid, orange line represents the linear dose-response modelled by random effect with restricted maximum likelihood methods. The solid black and dashed lines represent the non-linear dose-response and 95% CIs, respectively, modelled with restricted cubic splines.

## Supplemental Figure 42 – Dose response for the effect of substituting soymilk for cow’s milk on BMI

Individual trials are represented by the circles, with the weight of the comparison in the analysis represented by the size of the circle. The solid, orange line represents the linear dose-response modelled by random effect with restricted maximum likelihood methods. The solid black and dashed lines represent the non-linear dose-response and 95% CIs, respectively, modelled with restricted cubic splines.

## Supplemental Figure 43 – Publication bias funnel plot for the effect of substituting soymilk for cow’s milk on LDL-C

Contour-enhanced funnel plot is a scatterplot of each trial weighted mean difference on the x-axis with the standard error representing precision on the y-axis. The vertical solid red line represents the pooled effect estimate and the dashed red lines represent the pseudo-95% confidence limits. The blue dots represent individual trials. The contour regions define the regions for the test of significance of individual trial effect size for a given p-value range >0.100 (dark grey), 0.500 to <0.100 (medium grey), 0.010 to <0.500 (light grey), <0.0100 (white)]. The contour-enhanced funnel plots may suggest funnel-plot asymmetry is due to publication bias when less precise (smaller) trials are missing in the non-significant regions. Quantitative assessment of publication bias was also performed using Egger's and Begg's tests set at a significance level of p<0.100.

## SUPPLEMENTAL TABLES

## Supplemental Table 1 – PRISMA Checklist

| **Section/topic** | **#** | **Checklist item** | **Reported on page #** |  |  |
| --- | --- | --- | --- | --- | --- |
| **TITLE** | | |  |  |  |
| Title | 1 | Identify the report as a systematic review, meta-analysis, or both. | 1 |  |  |
| **ABSTRACT** | | |  |  |  |
| Structured summary | 2 | Provide a structured summary including, as applicable: background; objectives; data sources; study eligibility criteria, participants, and interventions; study appraisal and synthesis methods; results; limitations; conclusions and implications of key findings; systematic review registration number. | 2 |  |  |
| **INTRODUCTION** | | |  |  |  |
| Rationale | 3 | Describe the rationale for the review in the context of what is already known. | 3 |  |  |
| Objectives | 4 | Provide an explicit statement of questions being addressed with reference to participants, interventions, comparisons, outcomes, and study design (PICOS). | 3 |  |  |
| **METHODS** | | |  |  |  |
| Protocol and registration | 5 | Indicate if a review protocol exists, if and where it can be accessed (e.g., Web address), and, if available, provide registration information including registration number. | 2 (abstract) |  |  |
| Eligibility criteria | 6 | Specify study characteristics (e.g., PICOS, length of follow-up) and report characteristics (e.g., years considered, language, publication status) used as criteria for eligibility, giving rationale. | 3, Supplementary Table 3 and 5 |  |  |
| Information sources | 7 | Describe all information sources (e.g., databases with dates of coverage, contact with study authors to identify additional studies) in the search and date last searched. | 3 |  |  |
| Search | 8 | Present full electronic search strategy for at least one database, including any limits used, such that it could be repeated. | Supplementary Table 2 and 4 |  |  |
| Study selection | 9 | State the process for selecting studies (i.e., screening, eligibility, included in systematic review, and, if applicable, included in the meta-analysis). | 3 |  |  |
| Data collection process | 10 | Describe method of data extraction from reports (e.g., piloted forms, independently, in duplicate) and any processes for obtaining and confirming data from investigators. | 4 |  |  |
| Data items | 11 | List and define all variables for which data were sought (e.g., PICOS, funding sources) and any assumptions and simplifications made. | 4 |  |  |
| Risk of bias in individual studies | 12 | Describe methods used for assessing risk of bias of individual studies (including specification of whether this was done at the study or outcome level), and how this information is to be used in any data synthesis. | 4 |  |  |
| Summary measures | 13 | State the principal summary measures (e.g., risk ratio, difference in means). | 4 |  |  |
| Synthesis of results | 14 | Describe the methods of handling data and combining results of studies, if done, including measures of consistency (e.g., I^2^) for each meta-analysis. | 4 |  |  |
| Risk of bias across studies | 15 | Specify any assessment of risk of bias that may affect the cumulative evidence (e.g., publication bias, selective reporting within studies). | 4,5 |  |  |
| Additional analyses | 16 | Describe methods of additional analyses (e.g., sensitivity or subgroup analyses, meta-regression), if done, indicating which were pre-specified. | 4,5 |  |  |
| **RESULTS** | | |  |  |  |
| Study selection | 17 | Give numbers of studies screened, assessed for eligibility, and included in the review, with reasons for exclusions at each stage, ideally with a flow diagram. | 5,6, Figure 1 and Supplemental Figure 1 |  |  |
| Study characteristics | 18 | For each study, present characteristics for which data were extracted (e.g., study size, PICOS, follow-up period) and provide the citations. | 6, Table 1 and Supplemental Table 6 |  |  |
| Risk of bias within studies | 19 | Present data on risk of bias of each study and, if available, any outcome level assessment (see item 12). | 6, Supplemental Table 7 |  |  |
| Results of individual studies | 20 | For all outcomes considered (benefits or harms), present, for each study: (a) simple summary data for each intervention group (b) effect estimates and confidence intervals, ideally with a forest plot. | 7 and Supplemental Figures 2-20 |  |  |
| Synthesis of results | 21 | Present results of each meta-analysis done, including confidence intervals and measures of consistency. | 7 and Figure 2 |  |  |
| Risk of bias across studies | 22 | Present results of any assessment of risk of bias across studies (see Item 15). | 8 and Supplemental Figure 42 |  |  |
| Additional analysis | 23 | Give results of additional analyses, if done (e.g., sensitivity or subgroup analyses, meta-regression [see Item 16]). | 8 and Supplemental Figures 21-35 and Supplemental Table 8 |  |  |
| **DISCUSSION** | | |  |  |  |
| Summary of evidence | 24 | Summarize the main findings including the strength of evidence for each main outcome; consider their relevance to key groups (e.g., healthcare providers, users, and policy makers). | 8,9 and Supplemental Table 10 |  |  |
| Limitations | 25 | Discuss limitations at study and outcome level (e.g., risk of bias), and at review-level (e.g., incomplete retrieval of identified research, reporting bias). | 10 |  |  |
| Conclusions | 26 | Provide a general interpretation of the results in the context of other evidence, and implications for future research. | 10,11 |  |  |
| **FUNDING** | | |  |  |  |
| Funding | 27 | Describe sources of funding for the systematic review and other support (e.g., supply of data); role of funders for the systematic review. | 11 |  |  |

## Supplemental Table 2 – Search strategy for the effect of substituting soymilk for cow’s milk on intermediate cardiometabolic outcomes

| **MEDLINE**  **1946-May 5, 2023** | **EMBASE**  **1947-May 5, 2023** | **Cochrane Central Register of Controlled Trials**  **1991-May 5, 2023** |
| --- | --- | --- |
| 1. Milk/ 2. cow's milk.mp. 3. dairy milk.mp. 4. dairy beverage.mp. 5. dairy drink.mp. 6. milk beverage.mp. 7. bovine milk.mp. 8. exp cow's milk/ 9. (cow adj3 milk).mp. [mp=title, book title, abstract, original title, name of substance word, subject heading word, floating sub-heading word, keyword heading word, organism supplementary concept word, protocol supplementary concept word, rare disease supplementary concept word, unique identifier, synonyms] 10. or/1-9 11. Soy Milk/ 12. soy beverage.mp. 13. soy* milk.mp. 14. soya milk.mp. 15. soy drink.mp. 16. soy* drink.mp. 17. exp soy milk/ 18. (soy adj3 milk).mp. [mp=title, book title, abstract, original title, name of substance word, subject heading word, floating sub-heading word, keyword heading word, organism supplementary concept word, protocol supplementary concept word, rare disease supplementary concept word, unique identifier, synonyms] 19. (soy adj3 beverage).mp. [mp=title, book title, abstract, original title, name of substance word, subject heading word, floating sub-heading word, keyword heading word, organism supplementary concept word, protocol supplementary concept word, rare disease supplementary concept word, unique identifier, synonyms] 20. or/11-19 21. randomized controlled trial.pt. 22. (random$ or placebo$ or single blind$ or double blind$ or triple blind$).ti,ab. 23. (retraction of publication or retracted publication).pt. 24. or/21-23 25. (animals not humans).sh. 26. ((comment or editorial or meta-analysis or practice-guideline or review or letter) not "randomized controlled trial").pt. 27. (random sampl$ or random digit$ or random effect$ or random survey or random regression).ti,ab. not "randomized controlled trial".pt. 28. 24 not (25 or 26 or 27) 29. 10 and 20 and 28 | 1. Milk/ 2. cow's milk.mp. 3. dairy milk.mp. 4. dairy beverage.mp. 5. dairy drink.mp. 6. milk beverage.mp. 7. bovine milk.mp. 8. exp cow's milk/ 9. (cow adj3 milk).mp. [mp=title, abstract, heading word, drug trade name, original title, device manufacturer, drug manufacturer, device trade name, keyword heading word, floating subheading word, candidate term word] 10. or/1-9 11. Soy Milk/ 12. soy beverage.mp. 13. soy* milk.mp. 14. soya milk.mp. 15. soy drink.mp. 16. soy* drink.mp. 17. exp soy milk/ 18. (soy adj3 milk).mp. [mp=title, abstract, heading word, drug trade name, original title, device manufacturer, drug manufacturer, device trade name, keyword heading word, floating subheading word, candidate term word] 19. (soy adj3 beverage).mp. [mp=title, abstract, heading word, drug trade name, original title, device manufacturer, drug manufacturer, device trade name, keyword heading word, floating subheading word, candidate term word] 20. or/11-19 21. (random$ or placebo$ or single blind$ or double blind$ or triple blind$).ti,ab. 22. RETRACTED ARTICLE/ 23. or/21-22 24. (animal$ not human$).sh,hw. 25. (book or conference paper or editorial or letter or review).pt. not exp randomized controlled trial/ 26. (random sampl$ or random digit$ or random effect$ or random survey or random regression).ti,ab. not exp randomized controlled trial/ 27. 23 not (24 or 25 or 26) 28. 10 and 20 and 27 | 1. Milk/ 2. cow's milk.mp. 3. dairy milk.mp. 4. dairy beverage.mp. 5. dairy drink.mp. 6. milk beverage.mp. 7. bovine milk.mp. 8. exp cow's milk/ 9. (cow adj3 milk).mp. [mp=title, original title, abstract, floating sub-heading word, mesh headings, heading words, keyword] 10. or/1-9 11. Soy Milk/ 12. soy beverage.mp. 13. soy* milk.mp. 14. soya milk.mp. 15. soy drink.mp. 16. soy* drink.mp. 17. exp soy milk/ 18. (soy adj3 milk).mp. [mp=title, original title, abstract, floating sub-heading word, mesh headings, heading words, keyword] 19. (soy adj3 beverage).mp. [mp=title, original title, abstract, floating sub-heading word, mesh headings, heading words, keyword] 20. or/11-19 21. 10 and 20 22. limit 21 to (medline records and embase records) 23. 21 not 22 |

## Supplemental Table 3 – PICOTS table of the search strategy for the effect of substituting soymilk for cow’s milk on intermediate cardiometabolic outcomes

| PICOTS Framework defined in the main analysis of the present systematic review and meta-analysis | | | | | |
| --- | --- | --- | --- | --- | --- |
| Participants | Intervention | Comparator | Outcomes | Time | Study Design |
| Adults >18 years of age with all health statuses | Soymilk | Cow’s Milk | Established markers of blood lipids, glycemic control, blood pressure, inflammation, adiposity, renal function and structure, uric acid, and non-alcoholic fatty liver disease | >3 weeks | Randomized controlled trials done in humans |

*PICOTS, participants, interventions, comparators, outcomes, time, and study design

## Supplemental Table 4 – Search strategy for sub-study analysis on the effect of lactose vs. added sugars on intermediate cardiometabolic outcomes

| **MEDLINE**  **1946-June 7, 2023** | **EMBASE**  **1947-June 7, 2023** | **Cochrane Central Register of Controlled Trials**  **Through June 7, 2023** |
| --- | --- | --- |
| 1. exp Lactose/ 2. Lactose-containing.mp. 3. lactose powder.mp. 4. randomized controlled trial.pt. 5. controlled clinical trial.pt. 6. randomized.ab. 7. placebo.ab. 8. clinical trials as topic.sh. 9. randomly.ab. 10. trial.ti. 11. or/4-10 12. exp animals/ not humans.sh. 13. 11 not 12 14. or/1-3 15. 13 and 14 | 1. exp Lactose/ 2. Lactose-containing.mp. 3. lactose powder.mp. 4. 'randomized controlled trial'/ 5. 'controlled clinical trial'/ 6. 'random*'.ti,ab,tt. 7. 'randomization'/ 8. placebo.ti,ab,tt. 9. (compare or compared or comparison).ti,tt. 10. (evaluated or evaluate or evaluating or assessed or assess).ab. 11. (compare or compared or comparing or comparison).ab. 12. 10 and 11 13. (open adj label).ti,ab,tt. 14. ((double or single or doubly or singly) adj (blind or blinded or blindly)).ti,ab,tt. 15. double blind procedure/ 16. (parallel adj group*).ti,ab,tt. 17. (crossover or "cross over").ti,ab,tt. 18. ((assign* or match or matched or allocation) adj6 (alternate or group or groups or intervention or interventions or patient or patients or subject or subjects or participant or participants)).ti,ab,tt. 19. (assigned or allocated).ti,ab,tt. 20. (controlled adj8 (study or design or trial)).ti,ab,tt. 21. (volunteer or volunteers).ti,ab,tt. 22. human experiment/ 23. trial.ti,tt. 24. (or/4-9) or (or/12-23) 25. (random* adj sampl* adj8 ("cross section*" or questionnaire* or survey or surveys or database or databases)).ti,ab,tt. not (comparative study/ or controlled study/ or "randomised controlled".ti,ab,tt. or "randomized controlled".ti,ab,tt. or "randomly assigned".ti,ab,tt.) 26. cross-sectional study/ not (randomized controlled trial/ or controlled clinical study/ or controlled study/ or "randomised controlled".ti,ab,tt. or "randomized controlled".ti,ab,tt. or "control group".ti,ab,tt. or "control groups".ti,ab,tt.) 27. (("case control*" and random*) not ("randomised controlled" or "randomized controlled")).ti,ab,tt. 28. ("systematic review" not (trial or study)).ti,tt. 29. (nonrandom* not random*).ti,ab,tt. 30. "random field*".ti,ab,tt. 31. ("random cluster" adj4 sampl*).ti,ab,tt. 32. (review.ab. and review.pt.) not trial.ti,tt. 33. "we searched".ab. and (review.ti,tt. or review.pt.) 34. "update review".ab. 35. (databases adj5 searched).ab. 36. (rat or rats or mouse or mice or swine or porcine or murine or sheep or lambs or pigs or piglets or rabbit or rabbits or cat or cats or dog or dogs or cattle or bovine or monkey or monkeys or trout or marmoset*).ti,tt. and animal experiment/ 37. animal experiment/ not (human experiment/ or human/) 38. or/25-37 39. 24 not 38 40. or/1-3 41. 39 and 40 42. limit 41 to ("remove medline records" and (clinical trial or randomized controlled trial or controlled clinical trial or multicenter study)) | 1. exp Lactose/ 2. lactose-containing.mp. 3. lactose powder.mp. 4. or/1-3 5. limit 4 to (medline records and embase records) 6. 4 not 5 |

## Supplemental Table 5 – PICOTS table of the search strategy for the sub-study analysis on the effect of lactose vs. added sugars on intermediate cardiometabolic outcomes

| PICOTS Framework defined in the present systematic review and meta-analysis | | | | | |
| --- | --- | --- | --- | --- | --- |
| Participants | Intervention | Comparator | Outcomes | Time | Study Design |
| Adults >18 years of age with all health statuses | Added Sugars | Lactose | Established markers of blood lipids, glycemic control, blood pressure, inflammation, adiposity, renal function and structure, uric acid, and non-alcoholic fatty liver disease | >3 weeks | Controlled trials done in humans |

*PICOTS, participants, interventions, comparators, outcomes, time, and study design

## Supplemental Table 6 – Minimum Important Differences

| **Outcome** | **MID** | **References** |
| --- | --- | --- |
| Lipids (LDL-C, HDL-C, non-HDL-C, triglycerides) | 0.1 mmol/L*  *We used the MID identified as part of the development of the CCS dyslipidemia guidelines which was based on the health claim framework for cholesterol lowering and CHD risk reduction claims in Canada, US, and Europe, where the minimum reduction for permitted health claims was 3-5 % or 0.1 mmol/L | 1. Anderson TJ, Gregoire J, Pearson GJ, Barry AR, Couture P, Dawes M, et al. 2016 Canadian Cardiovascular Society Guidelines for the Management of Dyslipidemia for the Prevention of Cardiovascular Disease in the Adult. Can J Cardiol. 2016;32(11):1263-82.  2. Summary of Health Canada’s Assessment of a Health Claim about Soy Protein and Cholesterol Lowering Ottawa: Health Canada; 2015 [Available from: <https://www.canada.ca/en/health-canada/services/food-nutrition/food-labelling/health-claims/assessments/summary-assessment-health-claim-about-protein-cholesterol-lowering.html.>  3. Oat Products and Blood Cholesterol Lowering. Summary of Assessment of a Health Claim about Oat Products and Blood Cholesterol Lowering Ottawa: Health Canada; 2010 [Available from: <https://www.canada.ca/en/health-canada/services/food-nutrition/food-labelling/health-claims/assessments/summary-assessment-health-claim-about-protein-cholesterol-lowering.html.> |
| HbA1c | 0.3 % | European Medicines Agency. Guideline on clinical investigation of medicinal products in 4 the treatment or prevention of diabetes mellitus. 29 January 2018. CPMP/EWP/1080/00 Rev. 1. <https://www.ema.europa.eu/en/documents/scientific-guideline/draft-guideline-clinical-investigation-medicinal-products-treatment-prevention-diabetes-mellitus_en.pdf> |
| Fasting Glucose | 0.5 mmol/L | David M. Nathan, Judith Kuenen, Rikke Borg, Hui Zheng, David Schoenfeld, and Robert J. Heine, for the A1c-Derived Average Glucose (ADAG) Study Group. Diabetes Care 2008 <https://professional.diabetes.org/diapro/glucose_calc> |
| Fasting Insulin | 5 pmol/L*  *Proportional reduction to fasting glucose | - |
| CRP | 0.5 mg/L | 1. Reynolds Risk Score. Available at: http://www.reynoldsriskscore.org/Default.aspx [Accessed March 14, 2018].  2. Ridker PM, Paynter NP, Rifai N, Gaziano JM, Cook NR. C-reactive protein and parental history improve global cardiovascular risk prediction: the Reynolds Risk Score for men. Circulation. 2008;118(22):2243-51, 4p following 51.  3. Ridker PM, Buring JE, Rifai N, Cook NR. Development and validation of improved algorithms for the assessment of global cardiovascular risk in women: the Reynolds Risk Score. JAMA. 2007;297(6):611-9. |
| Blood Pressure (systolic and diastolic blood pressure) | 2 mmHg | Lewington S, Clarke R, Qizilbash N, Peto R, Collins R, Prospective Studies C. Age-specific relevance of usual blood pressure to vascular mortality: a meta-analysis of individual data for one million adults in 61 prospective studies. Lancet. 2002;360(9349):1903-13. |
| Body Weight | 0.5 kg | Ge L, Sadeghirad B, Ball GDC, da Costa BR, Hitchcock CL, Svendrovski A, et al. Comparison of dietary macronutrient patterns of 14 popular named dietary programmes for weight and cardiovascular risk factor reduction in adults: systematic review and network meta-analysis of randomised trials. BMJ. 2020;369:m696. |
| BMI* | 0.2 kg/m^2^*  *Approximately equivalent to 0.5 kg of body weight | - |
| Body Fat | 2 % | - |
| Waist Circumference | 2 cm*  *Assumption is made that a 1 kg change in body weight is related to a 1 cm change in waist circumference | Sacks FM, Bray GA, Carey VJ, Smith SR, Ryan DH, Anton SD, et al. Comparison of weight-loss diets with different compositions of fat, protein, and carbohydrates. N Engl J Med. 2009;360(9):859-73. |
| eGFR | 5 mL/min x 1.73m^2^/year | Kidney Disease: Improving Global Outcomes (KDIGO) CKD Work Group. KDIGO 2012 Clinical Practice Guideline for the Evaluation and Management of Chronic Kidney Disease. Kidney Int. Suppl. 2013(3):1–150. |
| Creatinine | 10 umol/L | Kidney Disease: Improving Global Outcomes (KDIGO) CKD Work Group. KDIGO 2012 Clinical Practice Guideline for the Evaluation and Management of Chronic Kidney Disease. Kidney Int. Suppl. 2013(3):1–150. |
| ALT | 2.85 IU/L | Tietz Textbook of Clinical Chemistry. Edited by CA Burtis, ER Ashwood. Philadelphia, WB Saunders Company, 1994 |
| AST | 2.55 IU/L | Tietz Textbook of Clinical Chemistry. Edited by CA Burtis, ER Ashwood. Philadelphia, WB Saunders Company, 1994 |

## Supplemental Table 7 - Trial characteristics for the main analysis

^a^HC: hypercholesterolemic; OW/OB: overweight or obesity; T2D: type 2 diabetic, PM: post-menopausal; RA: rheumatoid arthritis; HTN: hypertensive

^b^P = parallel design, C = crossover design

^c^OP = outpatient, IP = inpatient

^d^Cow’s milk lactose content assumed to be 12g/250 mL if the data was unreported.

^e^Skim: 0% milk fat, Low Fat: 1% milk fat, Reduced Fat: 1.5-2.5% milk fat, Whole: 3% Milk Fat

^f^Energy neutral background diet includes interventions designed to continue habitual caloric intake, energy reduced diets include inteventiorns designed to creatine a calorie deficit compared to the baseline diet, and protein limited diets include interventions where the participants’ habitual protein consumption has been decreased to accommodate the protein provided by the study foods

^g^Agency funding included government, not-for-profit health agencies or University sources

^h^Cells with a ‘-‘ represent unreported data

^i^Fortified (US definition: ‘fortified with calcium, vitamin A, and vitamin D’ [Dietary Guidelines for Americans, 2020-2025. In: U.S. Department of Agriculture and U.S. Department of Health and Human Services, editor. 9 ed2020.]): Y = Yes (explicitly states fortified soymilk was use, or suggests soymilk has comparable calcium, vitamin D, and vitamin A to that of cow’s milk), N = Non-fortified (explicitly states non-fortified soymilk was use, or suggests soymilk does not have comparable calcium, vitamin D, and vitamin A to that of cow’s milk), U = Unknown (no fortification information available)

## Supplemental Table 8 - Sensitivity analyses of the use of correlation coefficient of 0.25 and 0.75 for paired analysis in the analysis of the effect of substituting soymilk for cow’s milk on intermediate cardiometabolic outcomes

| **MD [95% CI], P_MD_, I^2^, P_Q_** | | | |
| --- | --- | --- | --- |
| **Outcome (10/17 trials were crossover)** | **Correlation coefficient used in the primary analysis** | **Correlation coefficient used in the sensitivity analysis** | |
|  | **0.5** | **0.25** | **0.75** |
| **Blood Lipids** | | | |
| LDL-C (mmol/L) | -0.19 [-0.29 to -0.09], P_MD_=<0.001, , I^2^= 0.00%, P_Q_=0.823 | -0.20 [-0.28 to -0.12], P_MD_=<0.001, I^2^= 0.00%, P_Q_=0.910 | -0.19 [-0.29 to -0.09], P_MD_=<0.001, I^2^= 0.00%, P_Q_=0.520 |
| HDL-C (mmol/L) | 0.05 [-0.09 to 0.00], P_MD_=0.036, I^2^= 0%, P_Q_=0.530 | 0.04 [-0.10 to 0.01], P_MD_=0.107, I^2^= 0%, P_Q_=0.670 | 0.05 [-0.10 to 0.01], P_MD_=0.089, I^2^= 0%, P_Q_=0.640 |
| Non-HDL-C (mmol/L) | -0.26 [-0.41 to -0.10], P_MD_=0.002, I^2^= 0.00%, P_Q_=0.963 | -0.24 [-0.42 to -0.06], P_MD_=0.008, I^2^= 0.00%, P_Q_=0.970 | -0.26 [-0.38 to -0.14], P_MD_=<0.001, I^2^= 0.00%, P_Q_=0.940 |
| Triglycerides (mmol/L) | -0.10 [-0.23 to 0.04], P_MD_==0.155, I^2^= 50.80%, P_Q_=0.039 | -0.08 [-0.22 to 0.06], P_MD_=0.246, I^2^= 41.41%, P_Q_=0.090 | -0.09 [-0.22 to 0.04], P_MD_=0.162, I^2^= 52.24%, P_Q_=0.030 |
| **Glycemic Control** | | | |
| HbA1c (%) | -0.37 [-1.13 to 0.39], P_MD_=0.337, I^2^=%,  P_Q_=. | -0.37 [-1.30 to 0.56], P_MD_=0.337, I^2^=%,  P_Q_=. | -0.37 [-0.91 to 0.17], P_MD_=0.176, I^2^=%,  P_Q_=. |
| Fasting Plasma Glucose(mmol/L) | 0.01 [-0.10 to 0.12], P_MD_=0.848, I^2^= 0.00%, P_Q_=0.669 | 0.01 [-0.12 to 0.14], P_MD_=0.800, I^2^= 0.00%, P_Q_=0.900 | 0.02 [-0.07 to 0.10], P_MD_=0.715, I^2^= 9.01%, P_Q_=0.360 |
| Fasting Insulin (umol/L) | 5.03 [-5.00 to 15.06],  P_MD_=0.326, I^2^= 60.98%, P_Q_=0.053 | 5.25 [-5.71 to 16.21],  P_MD_=0.348, I^2^= 60.92%, P_Q_=0.050 | 4.68 [-3.65 to 13.01],  P_MD_=0.271, I^2^= 61.07%, P_Q_=0.050 |
| 2-hour Plasma Glucose (mmol/L) | -0.22 [-0.97 to 0.53], P_MD_=0.560, I^2^=%,  P_Q_=. | -0.22 [-1.13 to 0.68], P_MD_=0.631, I^2^=%,  P_Q_=. | -0.22 [-0.77 to 0.32], P_MD_=0.424, I^2^=%,  P_Q_=. |
| **Blood Pressure** | | | |
| Systolic Blood Pressure (mmHg) | -8.00 [-14.89 to -1.11], P_MD_=0.023, I^2^= 86.89%, P_Q_=<0.001 | -7.99 [-15.45 to -0.52], P_MD_=0.036, I^2^= 85.80%, P_Q_=<0.000 | -8.01 [-14.01 to -2.01], P_MD_=0.009, I^2^= 88.89%, P_Q_=<0.001 |
| Diastolic Blood Pressure (mmHg) | -4.74 [-9.17 to -0.31], P_MD_=0.036, I^2^= 77.31%, P_Q_=0.001 | -4.71 [-7.06 to -2.36], P_MD_=<0.001, I^2^= 73.99%, P_Q_=0.000 | -3.56 [-5.19 to -1.92], P_MD_=<0.001, I^2^= 82.71%, P_Q_=0.000 |
| **Inflammation** | | | |
| CRP (mg/dL) | -0.81 [-1.26 to -0.37], P_MD_=<0.001, I^2^= 0%, P_Q_=0.814 | -0.82 [-1.28 to -0.36], P_MD_=<0.001, I^2^= 0%, P_Q_=0.830 | -0.80 [-1.22 to -0.38], P_MD_=<0.001, I^2^= 0%, P_Q_=0.770 |
| **Adiposity** | | | |
| Body Weight (kg) | -0.57 [-2.58 to 1.44], P_MD_=0.580, I^2^= 0.00%, P_Q_=0.99 | -0.48 [-2.87 to 1.90], P_MD_=0.691, I^2^= 0.00%, P_Q_=0.99 | -0.65 [-2.07 to 0.78], P_MD_=0.374, I^2^= 0.00%, P_Q_=0.98 |
| BMI (kg/m^2^) | -0.08 [-0.84 to 0.68], P_MD_=0.841, I^2^= 0.00%, P_Q_=0.99 | -0.05 [-0.92 to 0.82], P_MD_=0.907, I^2^= 0.00%, P_Q_=0.99 | -0.11 [-0.70 to 0.47], P_MD_=0.704, I^2^= 0.00%, P_Q_=0.99 |
| Body Fat (%) | 0.91 [-5.30 to 7.12], P_MD_=0.774, I^2^=%,  P_Q_=. | 0.91 [-5.30 to 7.12], P_MD_=0.774, I^2^=%,  P_Q_=. | 0.91 [-5.30 to 7.12], P_MD_=0.774, I^2^=%,  P_Q_=. |
| Waist Circumference (cm) | -0.71 [-2.87 to 1.44], P_MD_=0.517, I^2^= 0.00%, P_Q_=0.68 | -0.64 [-3.19 to 1.92], P_MD_=0.625, I^2^= 0.00%, P_Q_=0.76 | -0.80 [-2.38 to 0.78], P_MD_=0.320, I^2^= 0.00%, P_Q_=0.51 |
| **Renal Function and Structure** | | | |
| Creatinine (umol/L) | 0.00 [-0.04 to 0.04], P_MD_=0.969, I^2^=%,  P_Q_=. | 0.00 [-0.06 to 0.06], P_MD_=0.868, I^2^=%,  P_Q_=. | 0.00 [-0.03 to 0.03], P_MD_=0.959, I^2^=%,  P_Q_=. |
| eGFR (mL/min) | -1.09 [-11.62 to 9.44], P_MD_=0.839, I^2^=%,  P_Q_=. | -1.09 [-13.97 to 11.79], P_MD_=0.886, I^2^=%,  P_Q_=. | -1.09 [-8.58 to 6.40], P_MD_=0.775, I^2^=%,  P_Q_=. |
| **Non-Alcoholic Fatty Liver Disease** | | | |
| ALT (IU/L) | 17.38 [-20.45 to 55.21], P_MD_=0.368, I^2^=%,  P_Q_=. | 17.38 [-21.40 to 56.16], P_MD_=0.380, I^2^=%,  P_Q_=. | 17.38 [-19.47 to 54.23], P_MD_=0.355, I^2^=%,  P_Q_=. |
| AST (IU/L) | -1.12 [-4.46 to 2.22], P_MD_=0.511, I^2^=%,  P_Q_=. | -1.12 [-4.94 to 2.70], P_MD_=0.566, I^2^=%,  P_Q_=. | -1.12 [-3.89 to 1.65], P_MD_=0.429, I^2^=%,  P_Q_=. |

*CI, confidence interval; MD, mean difference

## Supplemental Table 9 - Adverse Events and Assessment of Acceptability

| **Study** | **Adverse Events and Assessment of Acceptability** |
| --- | --- |
| Sirtori et al. 1999 | The acceptability of the soymilk and cow’s milk products were excellent in and adequate in 3 of the 21 participants. 1 participant complained of significant side effects during the cow’s milk period, which the authors suggest was related to lactose intolerance. |
| Sirtori et al. 2002 | All 20 participants completed the trial without difficulty and tolerated both the soymilk and cow’s milk well. However, on a post hoc recall, a few participants reported gastrointestinal difficulties from the cow’s milk, but no side effects were reported in relation to the soymilk. |
| Ryan-Borchers et al. 2006 | Both the soymilk and cow’s milk were well tolerated overall. There were no dropouts related to the study beverages. |
| Mohammad-Shahi et al. 2016 | Both the soymilk and cow’s milk were well tolerated; however, 1 participant withdrew during the soymilk period due to gastrointestinal difficulties. |
| Miraghajani et al. 2012 | 2 of the 25 participants withdrew during the soymilk period due to the taste and gastrointestinal difficulties. |
| Hasanpour et al. 2023 | 2 of the 50 participants withdrew due to gastrointestinal difficulties related to soymilk consumption. |
| Onning et al. 1998 | Participants completed a sensory evaluation to assess appearance, consistency, flavour, and general impression of the soymilk and cow’s milk after 1 week of consumption, and again after 3 weeks. At the initial evaluation, cow’s milk received the highest score for appearance and smell, however the scores for both the soymilk and cow’s milk decreased during the test period, with soymilk scoring especially low after 3 weeks of consumption. |
| Nourieh et al. 2012 | 2 of the 24 participants withdrew during the study due to gastrointestinal difficulties. |
| Keshavarz et al. 2012 | 2 of the 24 participants withdrew during the study due to gastrointestinal difficulties. |

*Nine of the 17 trials reported adverse events and five reported acceptability of the study beverages. Trials not listed in the table did not report on adverse events or acceptability.

## Supplemental Table 10 - GRADE certainty of evidence assessment for the effect of substituting soymilk for cow’s milk on intermediate cardiometabolic outcomes

| **Outcome and trial (N)** | **Study design** | **GRADE Assessment** | | | | | |  | **Effect (MD [95% CI], P_MD_)** | **Certainty of Evidence** | **Magnitude of the Effect** |
| --- | --- | --- | --- | --- | --- | --- | --- | --- | --- | --- | --- |
|  |  | **Downgrades** | | | | | **Upgrades** |  |  |  |  |
|  |  | **ROB** | **Inconsistency** | **Indirectness** | **Imprecision** | **Publication bias** | **Dose response** |  |  |  |  |
| **Blood Lipids** | | | | | | | | | | | |
| **LDL-C (10)** | Randomized trials | Not serious | Not serious | Not serious | Not serious^cc^ | Not serious | None | ↔ | 0.19 mmol/L  [-0.29 to -0.09], P=<0001 | ⨁⨁⨁⨁ **High** | Small Important |
| **HDL-C (8)** | Randomized trials | Not serious | Not serious | Not serious | Serious^a^ | Not serious^aa^ | None | ↔ | 0.05 mmol/L  [-0.09 to 0.00] P=0.036 | ⨁⨁⨁◯ **Moderate** | Trivial |
| **Non-HDL-C (7)** | Randomized trials | Not serious | Not serious | Not serious | Not serious | Not serious^aa^ | None | ↔ | -0.26 mmol/L [-0.43 to 0.0], P=0.002 | ⨁⨁⨁⨁ **High** | Moderate |
| **Triglycerides (9)** | Randomized trials | Not serious | Not serious^b^ | Not serious | Serious^c^ | Not serious^aa^ | Dose response gradient^bb^ | ↔ | -0.10 mmol/L [-0.23 to 0.04], P=0.155 | ⨁⨁⨁◯ **Moderate** | No Effect |
| **Glycemic Control** | | | | | | | | | | | |
| **HbA1c (1)** | Randomized trials | Not serious | Not serious | serious^d^ | Serious^e^ | Not serious^aa^ | None | ↔ | -0.37 % [-1.13 to 0.39], P=0.337 | ⨁⨁◯◯ **Low** | No Effect |
| **Fasting Plasma Glucose (5)** | Randomized trials | Not serious | Not serious | Not serious | Not serious | Not serious^aa^ | None | ↔ | 0.01 mmol/L [-0.10 to 0.12]. P=0.848 | ⨁⨁⨁⨁ **High** | No Effect |
| **2-hour Plasma Glucose (1)** | Randomized trials | Not serious | Not serious | serious^f^ | Serious^g^ | Not serious^aa^ | None | ↔ | -0.22 mmol/L [-0.97 to 0.53], P=0.560 | ⨁⨁◯◯ **Low** | No Effect |
| **Fasting Insulin (4)** | Randomized trials | Not serious | Not serious^h^ | Not serious | Serious^i^ | Not serious^aa^ | None | ↔ | 5.03 pmol/L [-5.00 to 15.06], P=0.326 | ⨁⨁⨁◯ **Moderate** | No Effect |
| **Fasting Insulin (sweetened soymilk) (2)** | Randomized trials | Not serious | Not serious | Not serious | Serious^j^ | Not serious^aa^ | None | ↔ | 9.91 pmol/L [-1.01 to 20.82] | ⨁⨁⨁◯ **Moderate** | No Effect |
| **Fasting Insulin (unsweetened soymilk) (2)** | Randomized trials | Not serious | Not serious | Not serious | Serious^k^ | Not serious^aa^ | None | ↔ | -21.61 pmol/L [-47.10 to 3.89] | ⨁⨁⨁◯ **Moderate** | No Effect |
| **Blood Pressure** | | | | | | | | | | | |
| **Systolic Blood Pressure (5)** | Randomized trials | Not serious | Not serious^l^ | Not serious | Serious^m^ | Not serious^aa^ | None | ↔ | -8.00 mmHg [-14.89 to -1.11], P=0.023 | ⨁⨁⨁◯ **Moderate** | Moderate |
| **Diastolic Blood Pressure (5)** | Randomized trials | Not serious | Not serious^n^ | Not serious | Serious^o^ | Not serious^aa^ | None | ↔ | -4.74 mmHg [-9.17 to -0.31], P=0.036 | ⨁⨁⨁◯ **Moderate** | Moderate |
| **Inflammation** | | | | | | | | | | | |
| **CRP (5)** | Randomized trials | Not serious | Not serious | Not serious | Serious^p^ | Not serious^aa^ | None | ↔ | -0.81 mg/dL [-1.26 to -0.37]. P=<0.001 | ⨁⨁⨁◯ **Moderate** | Small Important |
| **Adiposity Markers** | | | | | | | | | | | |
| **Body Weight (6)** | Randomized trials | Not serious | Not serious | Not serious | Serious^q^ | Not serious^aa^ | None | ↔ | -0.57 kg [-2.58 to 1.44], P=0.580 | ⨁⨁⨁◯ **Moderate** | No Effect |
| **BMI (6)** | Randomized trials | Not serious | Not serious | Not serious | Serious^t^ | Not serious^aa^ | None | ↔ | -0.08 kg/m^2^ [-0.84 to 0.68], P=0.841 | ⨁⨁⨁◯ **Moderate** | No Effect |
| **Body Fat (1)** | Randomized trials | Not serious | Not serious | Serious^u^ | Not serious | Not serious^aa^ | None | ↔ | 0.91 % [-5.40 to 7.1], P=0.774 | ⨁⨁⨁◯ **Moderate** | No Effect |
| **Waist Circumference (3)** | Randomized trials | Not serious | Not serious | Not serious | Not serious | Not serious^aa^ | None | ↔ | -0.71 cm [-2.87 to 1.44], P=0.517 | ⨁⨁⨁⨁ **High** | No Effect |
| **Kidney Markers** | | | | | | | | | | | |
| **Creatinine (1)** | Randomized trials | Not serious | Not serious | Serious^d^ | Serious^v^ | Not serious^aa^ | None | ↔ | 0.00 umol/L [-0.02 to 0.02], P=0.932 | ⨁⨁◯◯ **Low** | No Effect |
| **eGFR (1)** | Randomized trials | Not serious | Not serious | Serious^d^ | Serious^w^ | Not serious^aa^ | None | ↔ | -1.09 mL/min [-11.62 to 9.44], P=0.839 | ⨁⨁◯◯ **Low** | No Effect |
| **NAFLD Markers** | | | | | | | | | | | |
| **ALT (1)** | Randomized trials | Not serious | Not serious | Serious^x^ | Serious^y^ | Not serious^aa^ | None | ↔ | 17.38IU/L [-20.45 to 55.21], P=0.368 | ⨁⨁◯◯ **Low** | No Effect |
| **AST (1)** | Randomized trials | Not serious | Not serious | Serious^x^ | Serious^z^ | Not serious^aa^ | None | ↔ | -1.12 IU/L [-4.46 to 2.22 IU/L], P=0.511 | ⨁⨁◯◯  **Low** | No Effect |

*Since all included trials were randomized or non-randomized controlled trials, the certainty of the evidence was graded as high for all outcomes by default and then downgraded or upgraded based on pre-specified criteria. Criteria for downgrades included risk of bias (downgraded if the majority of trials were considered to be at high risk of bias); inconsistency (downgraded if there was substantial unexplained heterogeneity [I^2^ ≥ 50%, P < 0.1]; indirectness (downgraded if there were factors absent or present relating to the participants, interventions, or outcomes that limited the generalizability of the results); imprecision (downgraded if the 95% confidence interval crossed the minimally important difference [MID] for harm set at 0.1mmol/L for blood lipids, 0.3% for HbA1c, 0.5mmol/L for plasma glucose, 5pmol/L for insulin, 0.5mg/dL for CRP, 2mmHg for systolic and diastolic blood pressure, 0.5 kg for body weight, 0.2kg/m^2^ for BMI, 2% for body fat, 2cm for waist circumference, X umol/L for creatinine, X mL/min for eGFR, 2.85 IU/L for ALT, and 2.55 IU/L for AST, and publication bias (downgraded if there is evidence of publication bias based on funnel plot asymmetry and/or significant Egger’s or Begg’s tests (P<0.1) with confirmation by adjustment by Duval and Tweedie trim-and-fill analysis).

** We did not downgraded for serious risk of bias in any analysis, since the majority of trials were rated as either low risk of bias for each domain and overall.
a. Downgraded for serious imprecision, as the 95% confidence interval (-0.06 to 0.11mmol/L) overlaps the MID for lipids which was set at 0.1mmol/L.

b. No downgrade for serious inconsistency as the presence of substantial heterogeneity (I2=54.00%, PQ=0.026) was explained by the removal of Onning et al. or Steele et al.

c. Downgraded for serious imprecision, as the 95% confidence interval (-0.24 to 0.03mmol/L) overlaps the MID for lipids which was set at 0.1mmol/L.

d. Downgraded for serious indirectness, as data from only 1 trial (N=25) was available and included in the comparison.

e. Downgraded for serious imprecision, as the 95% confidence interval (-1.13 to 0.39%) overlaps the MID for HbA1c which was set at 0.3%.

f. Downgraded for serious indirectness, as data from only 1 trial (N=28) was available and included in the comparison.

g. Downgraded for serious imprecision, as the 95% confidence interval (-0.22 to 0.63mmol/L) overlaps the MID for 2h-PG which was set at 0.5mmol/L.

h. No downgrade for serious inconsistency as the presence of substantial heterogeneity (I2=61.00%, PQ=0.053) was explained by the removal of Hasanpour et al.

i. Downgraded for serious imprecision, as the 95% confidence interval (-5.00 to 15.06umol/L) overlaps the MID for fasting insulin which was set at 5umol/L.

j. Downgraded for serious imprecision, as the 95% confidence interval (-1.01 to 20.82umol/L) overlaps the MID for fasting insulin which was set at 5umol/L.

k. Downgraded for serious imprecision, as the 95% confidence interval (-47.1 to 3.89 umol/L) overlaps the MID for fasting insulin which was set at 5umol/L.

l. No downgrade for serious inconsistency as the presence of substantial heterogeneity (I2=86.79%, PQ=0.00) was explained by the removal of Rivas et al..

m. Downgraded for serious imprecision, as the 95% confidence interval (-14.89 to -1.11mmHg) overlaps the MID for lipids which was set at 2mmHg.

n. No downgrade for serious inconsistency as the presence of substantial heterogeneity (I2=77.31%, PQ=0.00) was explained by the removal of Rivas et al..

o. Downgraded for serious imprecision, as the 95% confidence interval (-9.17 to -0.31mmHg) overlaps the MID for lipids which was set at 2mmHg.

p. Downgraded for serious imprecision, as the 95% confidence interval (-1.04 to 0.33mg/L) overlaps the MID for lipids which was set at 0.5mg/L.

q. Downgraded for serious imprecision, as the 95% confidence interval (-0.01 to 1.43kg) overlaps the MID for body weight which was set at 0.5kg.

r. Downgraded for serious imprecision, as the 95% confidence interval (-1.19 to 0.31kg) overlaps the MID for body weight which was set at 0.5kg.

s. Downgraded for serious imprecision, as the 95% confidence interval (-0.01 to 1.43kg) overlaps the MID for body weight which was set at 0.5kg.

t. Downgraded for serious imprecision, as the 95% confidence interval (-0.16 to 0.20kg/m2) overlaps the MID for fasting insulin which was set at 0.2kg/m2.

u. Downgraded for serious indirectness, as data from only 1 trial (N=43) was available and included in the comparison.

v. Downgraded for serious imprecision, as the 95% confidence interval (-0.04 to 0.04mmol/L) overlaps the MID for lipids which was set at 0.01mmol/L.

w. Downgraded for serious imprecision, as the 95% confidence interval (-11.62 to 9.44mL/min) overlaps the MID for lipids which was set at 10mL/min.

x. Downgraded for serious indirectness, as data from only 1 trial (N=24) was available and included in the comparison.

y. Downgraded for serious imprecision, as the 95% confidence interval (-20.45 to 55.21Iu/L) overlaps the MID for lipids which was set at 2.85Iu/L.

z. Downgraded for serious imprecision, as the 95% confidence interval (-4.46 to 2.22Iu/L) overlaps the MID for lipids which was set at 2.55Iu/L.

aa. publication bias could not be assessed as there were <10 studies available for these outcomes.

bb. we did not upgrade for the dose response gradient, as the relationship seen at lower doses of soy protein was lost at higher doses

cc. CI for LDL was rounded to MID of 0.1mmol/L based on clinical judgement and therefore, was not downgraded for imprecision.

*CI, confidence interval; MD, mean difference; ROB, risk of bias
